# Diurnal Dynamics of Gaseous and Dissolved Metabolites and Microbiota Composition in the Bovine Rumen

**DOI:** 10.3389/fmicb.2017.00425

**Published:** 2017-03-17

**Authors:** Henk J. van Lingen, Joan E. Edwards, Jueeli D. Vaidya, Sanne van Gastelen, Edoardo Saccenti, Bartholomeus van den Bogert, André Bannink, Hauke Smidt, Caroline M. Plugge, Jan Dijkstra

**Affiliations:** ^1^Top Institute Food and NutritionWageningen, Netherlands; ^2^Animal Nutrition Group, Wageningen University & ResearchWageningen, Netherlands; ^3^Laboratory of Microbiology, Wageningen University & ResearchWageningen, Netherlands; ^4^Laboratory of Systems and Synthetic Biology, Wageningen University & ResearchWageningen, Netherlands; ^5^Animal Nutrition, Wageningen Livestock ResearchWageningen, Netherlands

**Keywords:** volatile fatty acids, hydrogen, methane, linseed oil, dairy cow, bacteria, methanogenic archaea

## Abstract

Diurnal patterns of ruminal fermentation metabolites and microbial communities are not commonly assessed when investigating variation in ruminal CH_4_ production. The aims of this study were to monitor diurnal patterns of: (i) gaseous and dissolved metabolite concentrations in the bovine rumen, (ii) H_2_ and CH_4_ emitted, and (iii) the rumen microbiota. Furthermore, the effect of dietary inclusion of linseed oil on these patterns was assessed. Four rumen cannulated multiparous cows were used in a cross-over design with two 17 days periods and two dietary treatments: a control diet and a linseed oil supplemented diet [40% maize silage, 30% grass silage, 30% concentrate on dry matter (DM) basis for both diets; fat contents of 33 vs. 56 g/kg of DM]. On day 11, rumen contents were sampled for 10 h after morning feeding to profile gaseous and dissolved metabolite concentrations and microbiota composition. H_2_ and CH_4_ emission (mass per unit of time) was measured in respiration chambers from day 13 to 17. A 100-fold increase in ruminal H_2_ partial pressure (contribution to the total pressure of rumen headspace gases) was observed at 0.5 h after feeding. This peak was followed by a decline to basal level. Qualitatively similar patterns after feeding were also observed for H_2_ and CH_4_ emission, ethanol and lactate concentrations, and propionate molar proportion, although the opposite pattern was seen for acetate molar proportion. Associated with these patterns, a temporal biphasic change in the microbial composition was observed as based on 16S ribosomal RNA with certain taxa specifically associated with each phase. Bacterial concentrations (log_10_ 16S ribosomal RNA gene copies based) were affected by time, and were increased by linseed oil supplementation. Archaeal concentrations (log_10_ 16S ribosomal RNA gene copies based) tended to be affected by time and were not affected by diet, despite linseed oil supplementation decreasing CH_4_ emission, tending to decrease the partial pressure of CH_4_, and tending to increase propionate molar proportion. Linseed oil supplementation affected microbiota composition, and was most associated with an uncultivated Bacteroidales taxon. In summary, our findings support the importance of diurnal dynamics for the understanding of VFA, H_2_, and CH_4_ production.

## Introduction

The rumen is home to a complex microbial ecosystem that enables ruminants to degrade a wide variety of feed components and metabolites. In this ecosystem, hydrolytic and fermentative bacteria convert carbohydrate polymers to saccharide monomers and ferment these monomers into metabolites such as volatile fatty acids (VFA), CO_2_, and H_2_. Methanogenic archaea then produce CH_4_, primarily from CO_2_ and H_2_ (Morgavi et al., 2010). As CH_4_ emitted into the environment contributes to global warming, abatement of the production of this gas in ruminants is one of the main targets of greenhouse gas mitigation practices for the livestock industry (Hristov et al., 2013).

Variation in enteric CH_4_ production has often been related to diet composition. Best fit empirical models reported by Moraes et al. (2014) identified fat content as one of the key dietary variables in predicting enteric CH_4_ emissions of distinct cattle categories. In line with dietary fat content as a key predictor, Grainger and Beauchemin (2011) reported that a 10 g/kg dry matter (DM) increase in dietary fat decreased CH_4_ yield from cattle by 1 g/kg DM ingested. Although Grainger and Beauchemin (2011) did not find an effect of the type of fatty acid in the diet on the decrease in CH_4_ yield, Patra (2013) reported that C18:3 had marked inhibitory effect on CH_4_ emission compared with other dietary fatty acids. Variation in enteric CH_4_ production has also been predicted to vary with the type of dietary carbohydrates, the consequent molar proportions of VFA (primarily acetate, propionate and butyrate) produced and H_2_ yield. Such effects have been included in several mechanistic models (e.g., Mills et al., 2001; Bannink et al., 2010). Nevertheless, with these empirical and mechanistic approaches, the diurnal dynamics of rumen microbial metabolism has commonly been ignored when assessing rumen fermentation end products, despite peaks in VFA (Hatew et al., 2015), H_2_ and CH_4_ occurring shortly after feed consumption (Rooke et al., 2014).

In a recent theoretical study, Van Lingen et al. (2016) investigated the sensitivity of the NAD^+^/NADH ratio to H_2_ partial pressure (*P*_H_2__) in the rumen, and proposed the NAD^+^/NADH ratio, rather than *P*_H_2__ directly, as a key-controller of fermentation end products, because it contributes to the redox homeostasis. Bannink et al. (2006), who estimated coefficients for VFA molar proportions based on substrate fermentation, previously suggested that incorporation of cofactor dynamics may be of importance for representing VFA molar proportions in non-steady state conditions. Model predictions of CH_4_ produced, which is driven by the H_2_ yield associated with the VFA molar proportions, may also benefit from the incorporation of cofactor dynamics. Similar to fermentation end products, the rumen microbiota itself is also affected by time after feeding, with the concentration of viable rumen bacteria initially declining after feeding and then increasing (Leedle et al., 1982). Furthermore, the composition of metabolically active bacteria adherent to ruminally incubated forage has recently been shown to be biphasic in time (Huws et al., 2016). Little is known about how ruminal archaeal populations are affected by time after feeding, as in recent years more emphasis has been placed on the effect of diet composition and daily feed intake on ruminal archaea and CH_4_ emission.

Studies of *in vivo* diurnal patterns that report simultaneously dissolved metabolite concentrations (e.g., ethanol, VFA, and lactate) and partial pressures of H_2_, CO_2_, and CH_4_ in the rumen along with emissions of H_2_ and CH_4_ are limited, particularly in combination with microbiota composition analysis. An integrated approach may provide additional insight into rumen metabolic dynamics, and factors influencing the production of CH_4_. The aim of this study was therefore to monitor the diurnal patterns of H_2_ and CH_4_, dissolved metabolites and microbiota in the rumen, as well as H_2_ and CH_4_ emission, and assess whether the dietary inclusion of linseed oil affected these patterns.

## Methods and materials

### Experimental design, cows, diets, sampling, and measurements

The experiment was conducted at the animal research facilities of Wageningen University & Research (Wageningen, the Netherlands). All experimental procedures were approved by the Institutional Animal Care and Use Committee of Wageningen University & Research and carried out under the Dutch Law on Animal Experimentation.

Four rumen fistulated multiparous Holstein-Friesian cows (364 ± 20 days in milk, 22.0 ± 6.0 kg of milk/day, containing 4.54 ± 0.91% of fat and 4.03 ± 0.67% of protein; mean ± *SD*) were blocked in pairs according to lactation stage, parity, and milk production. Blocks were balanced over treatment sequence in a 2 × 2 crossover design with repeated measurements within each period. Cows were fed either a control diet (CON; 40% corn silage, 30% grass silage, and 30% concentrates on DM basis; crude fat content of 33 g/kg DM) or a diet for which the concentrate of the control diet was supplemented with linseed oil (LSO; proportions of corn silage, grass silage, and concentrates unchanged, crude fat content of 56 g/kg DM; concentrate ingredient composition is presented in Table S1). There were two experimental periods of 17 days each, and a 28 day washout period between the two experimental periods to prevent potential carryover effects. Cows were fed equal portions and milked twice daily (6 a.m. and 4 p.m.). Concentrate was in meal form and manually mixed into the roughage mixture at the moment of feeding.

Diets were supplied *ad libitum* during the first 8 days of each period to let the cows adapt to the treatment diets and for recording of the individual feed intake. From day 9 to 17, dry matter intake (DMI) within a block was restricted to 95% of the *ad libitum* DMI of the animal consuming the lowest amount of feed during days 5–8, while ensuring that cows never received <80% of their voluntary DMI. Samples of grass silage, corn silage, and both concentrates were obtained when fresh feed was prepared (i.e., twice weekly). Samples of grass silage and corn silage were obtained when fresh feed was prepared (i.e., twice weekly). One pooled sample of each of the concentrates was obtained and represented the whole experiment. These samples were stored at −20°C pending analyses. On day 11 of each period, 60 mL of rumen gas was sampled and feed left in the feeding bins was weighed at set time intervals (0, 0.5, 1, 1.5, 2, 2.5, 3, 4, 5, 6, 7, 8, 9, and 10 h after feeding), and 60 mL of rumen fluid was also sampled (0, 0.5, 1, 1.5, 2, 3, 4, 6, 8, and 10 h after feeding). Fistula lids were customized with a stopcock to sample rumen headspace gas, and a Teflon hose to sample rumen fluid. The Teflon hose was equipped with a perforated plastic tail that was wrapped in two layers of burlap with a pore size of 2 mm to separate fluid from particulate matter, and held at the ventral sac of the rumen with a 1.5 kg lead weight. Both gas and fluid samples were taken with a 60 mL BD Luer-Lok syringe. Gas samples were stored in N_2_ flushed under-pressure serum bottles and analyzed within 72 h after collection. Fluid samples were stored at −80°C and −20°C pending microbial and HPLC analysis, respectively, whereas pH was measured immediately after sampling.

### Housing and respiration chambers

From the start of every experimental period cows were housed in tie-stalls, and then from 3 p.m. on day 13 until 9 a.m. on day 17 the cows were housed in one of four respiration chambers for recording of gaseous emissions of H_2_ and CH_4_. In each chamber temperature was 16°C and relative humidity was 65%. The chambers were equipped with thin walls with windows, to allow audio-visual contact in order to minimize the effect of social isolation on cow behavior and performance. Cows were exposed to 16 h of light per day, from 5.30 a.m. to 9.30 p.m. The ventilation rate within each chamber was 58 m^3^/h to ensure that the H_2_ peak after feeding was within reach of the H_2_ analyzer (i.e., 0–100 ppm). Exhaust air of the four chambers was sampled at 12 min intervals. Every fifth interval was increased to 15 min for sampling of the inlet air. A H_2_ gas analyzer with an electro chemical cell (MGA3000, ADC Gas Analysis Ltd, Hoddesdon, England, UK) was setup in series with the O_2_-, CO_2_-, and CH_4_-analyzers to determine the H_2_-concentration in sampled air. Gas concentrations and ventilation rates were corrected for pressure, temperature, and humidity to arrive at standard temperature pressure dew point volumes of inlet and exhaust air. Calibration gases were sampled for analysis instead of the inlet air once per day. The analyzed and actual values of these calibration gases were used to correct the measured gas concentrations from the inlet air and exhaust air of all compartments. Before the present experiment started, chambers were checked by releasing known amounts of CO_2_ in each compartment and comparing these values with the data from the gas analysis system to calculate the recovery, with recovered amounts being between 99 and 101%. All other aspects of the experimental setup of the respiration chambers were as described by van Gastelen et al. (2015), except for the fact that gas measurements during milking and feeding were retained in the dataset.

### Feed composition determination

Prior to analysis, feed samples were prepared as described by Hatew et al. (2015) and oven dried at 60°C, except for the ammonia analysis in the silages for which fresh samples were used. Dried feeds were analyzed for DM, neutral detergent fiber (NDF), acid detergent fiber (ADF), acid detergent lignin (ADL), ash, N (crude protein content calculated as N × 6.25), starch, sugars, and gross energy (GE) as described by Hatew et al. (2015), and for crude fat based on NEN-ISO 1735 (ISO 1735, 2004) with modifications as described by Klop et al. (2017).

### Analysis of concentrations of gaseous and dissolved metabolites

Gaseous metabolites were separated with a Compact GC gas chromatograph (Global Analyzer Solutions, Breda, The Netherlands) containing two lines. One line, which contained a Carboxen 1010 pre-column (Supelco, 3 m × 0.32 mm) followed by a Molsieve 5A column (Restek, 25 m × 0.32 mm), was used for H_2_ analysis. The following settings were applied: He carrier gas, 200 kPa pressure, 20 mL/min split flow rate and an oven temperature of 90°C. A Pulsed Discharge Detector held at 110°C was used for quantification. The other line, which contained a single Carboxen 1010 column (Supelco, 15 m × 0.32 mm), was used for detection of CO_2_ and CH_4_. This column had the following settings: He carrier gas, 200 kPa pressure, 10 mL/min split flow rate and an oven temperature of 80°C. A thermal conductivity detector held at 110°C was used for quantification.

Rumen fluid samples were centrifuged (10,000 g for 14 min) after which the metabolites dissolved in the supernatants were separated by a Spectrasystem HPLC (Thermo Scientific, Breda) equipped with a Metacarb 67H column (Agilent, 300 × 65 mm). Column temperature was 45°C, except for the determination of ethanol that was performed at 25°C. A 5 mM sulfuric acid solution was used as an eluent. Flow rate was set at 0.8 mL/min. Metabolites were quantified with a Refractive Index detector. Minimum detectable concentrations of ethanol and lactate were 0.74 and 0.25 mM, respectively. Total VFA concentration was calculated as the sum of the concentrations of acetate, propionate, butyrate, valerate, and isovalerate.

### DNA extraction

For performing quantitative PCR (qPCR) analysis for the quantification of total bacterial and archaeal concentrations, total genomic DNA (gDNA) was extracted from rumen fluid samples using a protocol involving a combination of bead beating, Stool Transport, and Recovery (STAR) buffer (Roche Diagnostics Nederland BV, Almere, The Netherlands) and the Maxwell® 16 Instrument (Promega, Leiden, The Netherlands). The method was developed from the previously described method of Salonen et al. (2010) by (i) changing the repeated bead beating buffer to the STAR buffer and then (ii) proceeding with the lysate directly into a customized Maxwell® 16 Tissue LEV Total RNA Purification Kit cartridge (XAS 1220). Briefly, cells were pelleted by centrifugation at 15,000 g for 10 min at 4°C from 1 mL of rumen fluid, resuspended in 700 μL of STAR buffer and transferred to a sterile screw-capped 2 mL tube (BIOplastics BV, Landgraaf, The Netherlands) containing 0.5 g of zirconium beads (0.1 mm; BioSpec Products, Inc., Oklahoma, USA) and 5 glass beads (2.5 mm; BioSpec Products). The sample was then treated in a bead beater (Precellys 24, Bertin technologies, Montigny-le-Bretonneux, France) at a speed of 5.5 m/s for 3 × 1 min, followed by incubation at 95°C with agitation (15 min and 300 rpm). The lysis tube was then centrifuged (13,000 g for 5 min at 4°C), and the supernatant transferred to a 2 mL microcentrifuge tube. STAR buffer (300 μL) was added to the remaining contents of the lysis tube, and all the previous steps starting with bead-beating repeated again. An aliquot (250 μL) of the combined supernatants from the sample lysis was then transferred into the custom Maxwell® 16 Tissue LEV Total RNA Purification Kit cartridge. The remainder of the extraction protocol was then carried out in the Maxwell® 16 Instrument according to the manufacturer's instructions. The quantity and purity of the resulting DNA was assessed using a NanoDrop ND-1000 spectrophotometer (NanoDrop® Technologies, Wilmington, DE, USA).

### RNA extraction and cDNA synthesis

RNA was extracted for use as a template for rumen microbiota composition analysis. This was due to (i) its ability to reflect the more metabolically active microbes and (ii) its more rapid degradation, relative to DNA, increasing the ability to assess differences in community composition occurring between relatively short (<1 h) time point intervals. As with the DNA extracts, cells were pelleted by centrifugation at 15,000 g for 10 min at 4°C from 1 mL of rumen fluid. The cell pellet was resuspended in 500 μL Tris-EDTA buffer (Tris-HCl pH 7.6, EDTA pH 8.0). Total RNA was extracted from the resuspended pellet according to the Macaloid-based RNA isolation protocol (Zoetendal et al., 2006) with the use of Phase Lock Gel heavy (5 Prime GmbH, Hamburg, Germany) during phase separation. The aqueous phase was purified using the RNAeasy mini kit (QIAGEN Benelux BV, Venlo, The Netherlands), including an on-column DNAseI (Roche) treatment as described previously (Zoetendal et al., 2006). Total RNA was eluted in 30 μL Tris-EDTA buffer. RNA quantity and quality were assessed using a NanoDrop ND-1000 spectrophotometer and an Experion RNA StdSens analysis kit (Bio-Rad Laboratories BV, Veenendaal, The Netherlands) respectively. Absence of contaminating DNA was confirmed by performing a PCR directly on the RNA extract using the first step PCR of the Universal 16S rRNA gene MiSeq protocol (see Section qPCR). Subsequently, total RNA (2.5 μg) was reverse transcribed using random hexamer primers with the Maxima H Minus First Strand cDNA synthesis kit (Fisher Scientific, Landsmeer, The Netherlands) following the manufacturer's guidelines. Non-template control reactions were also performed. cDNA preparations and control reactions were cleaned using a DNA Clean & Concentrator-5 kit (Zymo Research Europe GmbH, Freiburg, Germany) according to the manufacturers protocol.

### qPCR

For absolute quantification of bacteria and archaea, SYBR green qPCR assays were performed with sample DNA extracts using an iCycler iQ real-time detection system (Bio-Rad Laboratories BV). All qPCR analyses were carried out in triplicate with a reaction volume of 10 μL, using optical-grade PCR plates and sealing film. The reaction mixture contained 2 × iQ SYBR green PCR mixture (Bio-Rad Laboratories B.V.), 200 nM (final concentration) of each primer (Table 1), and 2 μL of either the DNA template or PCR grade water. The bacterial amplification program consisted of an initial denaturation at 94°C for 10 min followed by 35 cycles of 94°C for 20 s, 60°C for 30 s, and 72°C for 30 s. The archaeal amplification program consisted of an initial denaturation at 94°C for 10 min followed by 40 cycles of 94°C for 10 s, 60°C for 30 s, and 72°C for 30 s. The fluorescent products were detected at the last step of each cycle. Following amplification, melting temperature analysis of PCR products was performed to determine the specificity of the PCR. The melting curves were obtained by slow heating at 0.5°C/s increments from 60 to 95°C, with continuous fluorescence collection. Standard curves (10^2^–10^8^ amplicon copies/μL) for the assays were prepared using purified PCR amplicons amplified from gDNA of *Ruminococcus albus* (bacterial qPCR standard) and *Methanosarcina mazei* (archaeal qPCR standard) with the primers and annealing temperatures indicated in Table 1.

**Table 1:** **16S rRNA targeted primers and annealing temperatures (T_m_) used in this study**.

**Application**	**Primer^a^**	**Primer sequence (5′−3′)^b^**	**T_m_ (°C)^c^**	**Reference**
Bacterial qPCR standard	27F	AGAGTTTGATCCTGGCTCAG	55	Lane, 1991
	PROK1492R	GGWTACCTTGTTACGACTT		Suzuki et al., 2000
Archaeal qPCR standard	25F	CYGGTTGATCCTGCCRG	52	Dojka et al., 1998
	PROK1492R	GGWTACCTTGTTACGACTT		Suzuki et al., 2000
Bacterial qPCR	Bact1369F	CGGTGAATACGTTCYCGG	60	Suzuki et al., 2000
	PROK1492R	GGWTACCTTGTTACGACTT		
Archaeal qPCR	Arch-787f	ATTAGATACCCSBGTAGTCC	60	Yu et al., 2005
	Arch-1059r	GCCATGCACCWCCTC		
Universal 16S MiSeq	515f	GTGYCAGCMGCCGCGGTAA	–	Walters et al., 2015
	806rB	GGACTACNVGGGTWTCTAAT		
	UniTag1-515f	GAGCCGTAGCCAGTCTGCGTGYCAGCMGCCGCGGTAA^d^		This study
	UniTag2-806rB	GCCGTGACCGTGACATCGGGACTACNVGGGTWTCTAAT^d^		This study

a *Primer names may not correspond to the original publication*.

b *Degenerate nucleotides are described using the IUPAC nucleotide code*.

c *Annealing temperature used with the respective primer pairs*.

d *UniTag sequences are underlined*.

### Microbial composition analysis

For 16S rRNA based microbial composition profiling, barcoded amplicons from the V4 region of 16S rRNA were generated from cDNA using a 2-step PCR strategy. PCRs were performed with a SensoQuest Labcycler (Göttingen, Germany) using an adaptation of the cycling conditions of Walters et al. (2015) due to the use of the 2-step protocol (Tian et al., 2016) and the Phusion enzyme. The first PCR step was performed in a total volume of 50 μL containing 1 × HF buffer (Finnzymes, Vantaa, Finland), 1 μL dNTP Mix (10 mM; Promega), 1 U of Phusion® Hot Start II High-Fidelity DNA polymerase (Finnzymes), 500 nM each of the primers UniTag1-515f and UniTag2-806rB (Table 1) and 10–20 ng of sample cDNA. The cycling conditions for the first step consisted of an initial denaturation at 98°C for 3 min, 25 cycles of: 98°C for 10 s, 50°C for 20 s, and 72°C for 20 s, and a final extension at 72°C for 10 min. The size of the PCR products (~330 bp) was confirmed by agarose gel electrophoresis on a 2% (w/v) agarose gel containing 1 × SYBR® Safe (Invitrogen, Carlsbad, CA, USA).

The second PCR step was then employed to add an eight nucleotide sample specific barcode to the 5′- and 3′-end of the PCR products. This step was performed as previously described by Tian et al. (2016). Incorporation of the sample specific barcodes, yielding a PCR product of ~350 bp, was confirmed by agarose gel electrophoresis. Control PCR reactions were performed alongside each separate amplification with (i) the non-template control from the cDNA preparation and (ii) no addition of template, and consistently yielded no product. PCR products were then purified using HighPrep™ (MagBio Europe Ltd, Kent, United Kingdom) and quantified using a Qubit in combination with the dsDNA BR Assay Kit (Invitrogen). Purified PCR products were mixed in equimolar amounts into pools together with defined synthetic mock communities which allow assessment of potential technical biases (Ramiro-Garcia et al., 2016). Pools then underwent adaptor ligation followed by sequencing on the HiSeq platform with addition of 20% PhiX (GATC-Biotech, Konstanz, Germany).

The 16S rRNA cDNA gene sequencing data was then analyzed using NG-Tax, an in-house pipeline (Ramiro-Garcia et al., 2016). Paired-end libraries were filtered to contain only read pairs with perfectly matching barcodes, and those barcodes were used to demultiplex reads by sample. Operational taxonomic units (OTUs) were defined using an open reference approach, and taxonomy was assigned to those OTUs using a SILVA 16S rRNA gene reference database (Quast et al., 2013). The 16S rRNA sequence data generated in this study is deposited in the European Nucleotide Archive under the study accession number PRJEB17837.

### Statistical analysis

#### Metabolites and microbes in the rumen

Prior to statistical analyses, values of lactate concentration, *P*_H_2__ and bacterial and archaeal concentrations and the ratio of archaeal to bacterial concentrations, were log_10_-transformed. If a boxplot identified an outlier that could be related to the feed intake pattern of a cow, data points were removed. Gaseous and dissolved metabolite and microbial concentrations and pH in the rumen were subjected to a repeated-measures ANOVA using the following model:

(1) $$y_{ijkl}=\mu +\tau_{i}+\delta_{j}+\tau_{i}\delta_{j}+\pi_{k}+\gamma_{l}+e_{ijkl},$$

where *y*_*ijkl*_ represents the measurement from cow *l* at sampling number *i* given treatment *j* at period *k*; μ represents the overall mean; τ_*i*_ represents fixed effect of the *i*th sampling moment, *i* = 1, 2, …, 14 for rumen gases and pH, *i* = 3, 4, 5, 6 for lactate concentration, *i* = 2, 3, …, 6 for ethanol concentration, and *i* = 1, 2, …, 10 for all other dissolved metabolite and microbial concentrations; δ_*j*_ and π_*k*_ represent the fixed effects of diet (*j* = 1, 2) and period (*k* = 1, 2), respectively; γ_*l*_ represents random effect of cow (*l* = 1, 2, 3, 4); *e*_*ijkl*_ represents the residual error. With this non-repeated crossover design, potential carryover or residual effects due to the diet fed in the preceding period cannot be identified (Tempelman, 2004), and therefore no sequence effect was included in the model. Correlations of repeated measurements within period, fitted to a cow × period interaction, were modeled with a spatial power, exponential or spherical matrix structure. In case of non-positive definite random-effect or residual covariance matrix, either the random effect of cow and/or the spatial correlation structure were removed from the model. Matrix structure was evaluated using Akaike information criterion (AIC). Degrees of freedom were estimated using the Kenward-Roger approximation. Multiple comparisons were performed according to the Tukey-Kramer method. Data from sampling times with <5 values above the minimum detectable concentration were excluded from the analysis, which applied to lactate and ethanol concentrations. Analyses were carried out using PROC GLIMMIX in SAS (SAS Institute Inc., 2008). All results are reported as least squares means. Significance of effects was declared at *P* ≤ 0.05 and tendencies to significance at 0.05 < *P* ≤ 0.10.

#### Gaseous emissions

Translocation of cows to chambers may affect the gas emission profile of that particular day and therefore only data obtained between morning feedings on day 14 and 17 were evaluated. Since cows were fed at 10 and 14 h intervals every day, values of H_2_ and CH_4_ emission observed between morning and evening feeding, and evening and morning feeding were fitted to time separately. Values of H_2_ emission rate were log_10_-transformed to stabilize variance. Gas emission rates were evaluated using the following double-exponential and hyperbolic nonlinear models:

$$\begin{array}{l} y_{ijk}=\{\begin{array}{c} \phi_{1ij}+\phi_{2ij}\left(-{exp}^{\phi_{3ij}t}+{exp}^{\phi_{4ij}t}\right)+e_{ijk}\\ \phi_{1ij}+\frac{\phi_{2ij}t^{\phi_{4ij}}}{1+\phi_{3ij}t^{1+\phi_{4ij}}}+e_{ijk} \end{array},\\ \;\phi_{ij}=\left[\begin{array}{c} \phi_{1ij}\\ \phi_{2ij}\\ \phi_{3ij}\\ \phi_{4ij} \end{array}\right]=\left[\begin{array}{c} \beta_{1}\\ \beta_{2}\\ \beta_{3}\\ \beta_{4} \end{array}\right]+\left[\begin{array}{c} b_{1i}\\ b_{2i}\\ b_{3i}\\ b_{4i} \end{array}\right]+\left[\begin{array}{c} b_{1i,j}\\ b_{2i,j}\\ b_{3i,j}\\ b_{4i,j} \end{array}\right]=\beta +b_{i}+b_{i,j}, \end{array}$$

with

(2) $$b_{i}\sim N\left(0,\Psi_{1}\right),b_{i,j}\sim N\left(0,\Psi_{2}\right)\;and\;e_{ijk}\sim \{\begin{array}{c} N\left(0,\sigma^{2}\right)\\ N\left(0,\sigma^{2}{\left|\upsilon_{ijk}\right|}^{2\omega }\right)\\ N\left(0,\sigma^{2}{exp}^{2\omega \upsilon }\right) \end{array},$$

where **β** is the vector of fixed effects, where β_1_ is the asymptote, β_2_ is a linear multiplier, β_3_ and β_4_ represent the increase and decline of gas emission after feeding, respectively; ***b**_i_* is the vector of random effects of the cow × period interaction, with *i* = 1, …, 8 and its covariance matrix Ψ_1_; ***b***_*i,j*_ is the vector of random effects of portion nested within the cow × period interaction, with *j* = 1, 2, 3, and its covariance matrix Ψ_2_; *e*_*ijk*_ is the residual error with variance covariate υ_*ijk*_ (gas emission rate for the power function, gas emission rate or time from feeding for the exponential function) and unrestricted parameter ω (i.e., may take any real value, the variance increases or decreases with the variance covariate). Effect of dietary treatment on emission profile was evaluated by stepwise replacement of the four fixed-effects parameters (β_1_…, β_4_) according to:

(3) $$\beta_{n}=\delta_{n1}x_{n1}+\delta_{n2}x_{n2},$$

with $\left[\begin{array}{c} x_{n1}\\ x_{n2} \end{array}\right]=\left[\begin{array}{c} 1\\ 0 \end{array}\right]$ if diet is control and $\left[\begin{array}{c} x_{n1}\\ x_{n2} \end{array}\right]=\left[\begin{array}{c} 0\\ 1 \end{array}\right]$ if diet is linseed, and δ_*n*1_ and δ_*n*2_ the control and linseed diet main effects, respectively. Inclusion of treatment fixed effect and random effects, and random-effects covariance structure and residual variance were modeled using AIC. Model parameters for control and linseed diet were compared using Tukey's pairwise comparison. Analyses were carried out using *nlme* (Pinheiro and Bates, 2000) and *multcomp* (Hothorn et al., 2008) packages in R statistical software.

#### Microbiota composition

Microbial composition summary plots and Principal Coordinate Analysis (PCoA) of the weighted unifrac distance matrix of the OTU was performed using a workflow based on Quantitative Insights Into Microbial Ecology (QIIME) v1.2 (Caporaso et al., 2010). Permutational Multivariate Analysis of Variance (PERMANOVA; Anderson, 2001) was used to assess the significance of changes in the rumen microbiota composition with respect to the factors: time (10 levels), diet (2 levels: CON and LSO) and the factor interaction period × diet (4 levels). PERMANOVA was also used to test the effect of time by categorizing time points based on the concentration of rumen metabolites being either “high” (0.5–4 h; total concentration of VFA+lactate+ethanol ≥ 90 mM or a maximum in gas partial pressure) or “low” (0, 6–10 h; every other case). PERMANOVA and Bonferroni corrected multiple comparisons were applied on the weighted unifrac distance matrix using the Matlab Fathom toolbox (Jones, 2015). Redundancy analysis (RDA) was performed using Canoco 5 (Šmilauer and Leps, 2014) to assess the relationship between genus-level phylogenetic groupings of the OTU and time or diet.

## Results

### Composition of diets and feed intake

The composition of the grass silage, corn silage and the concentrates as well as total mixed ration is shown in Table 2. On day 11 of both experimental periods, cows started ingesting their portions immediately after morning feed delivery with the highest intake consistently occurring during the first 0.5 h after feeding (Figure S1). Small differences between cows in the time taken to finish their portions were observed, particularly with cow 2 in period 1 which took longer to finish its portion (8 h) compared to the other cows (2–6 h). Portion size, which is half of the daily DMI, was 9.1 ± 0.2 kg of DM and no refusals were found from any of the cows. During the chamber measurement days, 9.0 ± 0.3 kg of DM of the portions were ingested and feed refusals (0.1 ± 0.2 kg of DM) only occurred with cow 2.

**Table 2:** **Analyzed composition of grass silage, corn silage, and treatment concentrates [without linseed oil (CON) and with linseed oil (LSO)] and calculated composition of total mixed diets [g/kg dry matter (DM), unless stated otherwise]**.

	**Silage**		**Concentrate^b^**		**Dietary Treatment**	
**Item**	**Grass**	**Corn**	**CON**	**LSO**	**CON**	**LSO**
DM (g/kg)	554	316	878	890	465	466
Crude ash	91	41	120	111	79	77
Crude protein	140	80	394	361	192	182
Crude fat	30	35	33	108	33	56
NDF	542	333	203	178	357	349
ADF	322	202	101	91	208	205
ADL	15	9	17	16	13	13
Starch	ND^a^	373	18	14	154	153
Sugars	89	ND^a^	137	124	68	64
Gross Energy (MJ/kg of DM)	18.3	18.3	18.0	19.7	18.2	18.7

a *Not determined*.

b *For concentrate ingredient composition see Table S1*.

### Headspace gases, dissolved metabolites, and microbial numbers

**Figure 1:**
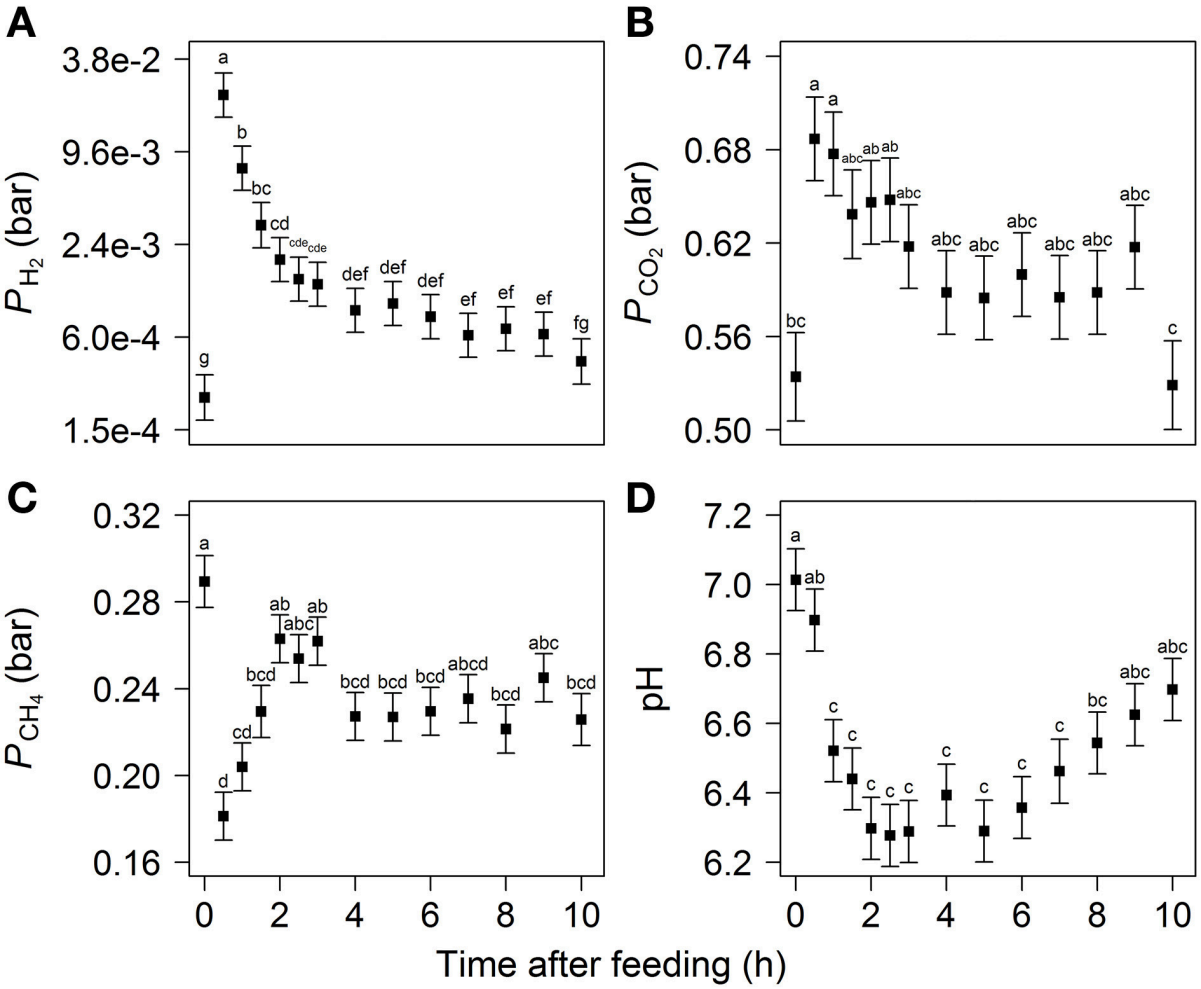
**Partial pressure of (A)** H_2_, **(B)** CO_2_, **(C)** CH_4_ in the rumen headspace, and **(D)** pH of rumen fluid over the first 10 h after feeding. Values represent least square mean (LSM) ± standard error, with different letters indicating significant differences in time (*P* < 0.05).

In response to feeding, *P*_H_2__ increased from 2.4 · 10^−4^ to 2.2 · 10^−2^ bar in 0.5 h and then steadily decreased to and did not significantly differ from the 0 h level at 10 h (Figure 1). A similar pattern was observed for *P*_CO_2__ which increased from 0.54 to 0.69 bar during the first 0.5 h and then decreased and did not differ from the 0 h level from 3 h onwards, with the numerically lowest *P*_CO_2__ of 0.53 bar at 10 h. The profile of *P*_CH_4__, however, showed a decrease from 0.29 to 0.18 bar over the first 0.5 h and then increased to values not different from 0 h level at 2, 2.5, 3, 7, and 9 h. From 4 to 10 h, *P*_CH_4__ was between 0.22 and 0.25 bar and did not significantly differ from the values observed at 2, 2.5, and 3 h. In contrast to *P*_H_2__ and *P*_CO_2__, which were not affected by diet (Table S2), *P*_CH_4__ tended to be lower for cows fed the linseed diet (2.4 · 10^−1^ ± 4.8 · 10^−3^ bar for CON vs. 2.3 · 10^−1^ ± 4.8 · 10^−3^ bar for LSO). No time × diet interaction was observed for any of the gaseous metabolites (*P* > 0.567). Rumen fluid pH was 7.0 at feeding, decreased to 6.3 by 2 h, remained relatively constant until 5 h and then increased to 6.7 at 10 h (Figure 1). The largest decrease was between 0.5 and 1 h and pH-values were significantly different from 0 h level from 1 to 8 h. Rumen fluid pH was not affected by diet (*P* = 0.538) and no time × diet interaction was observed (*P* = 0.902).

**Figure 2:**
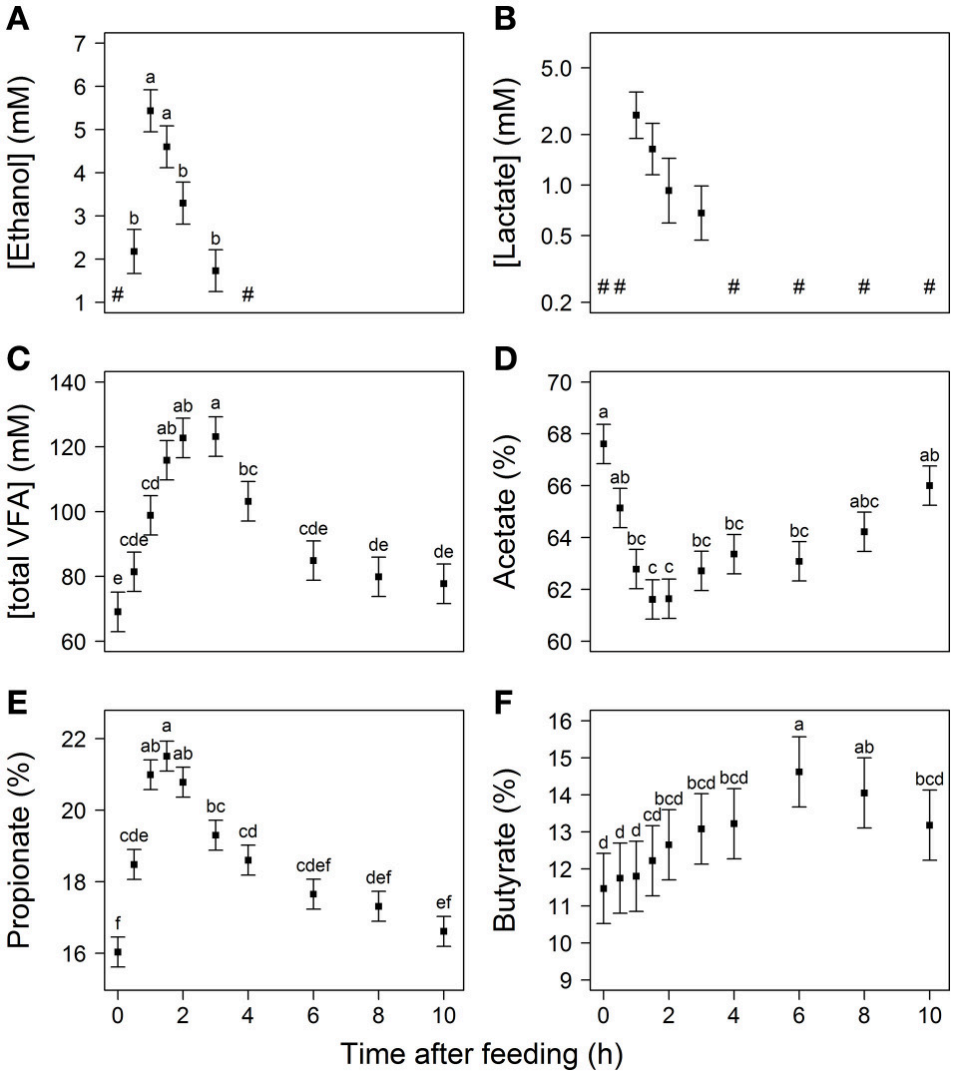
**Concentrations of (A)** ethanol, **(B)** lactate, **(C)** total VFA, and proportions of **(D)** acetate, **(E)** propionate and **(F)** butyrate in rumen fluid over the first 10 h after feeding. The “#” indicates that the metabolite concentration was non-detectable. Values represent least square mean (LSM) ± standard error, with different letters indicating significant differences in time (*P* < 0.05). Values of lactate concentration are back-transformed and plotted on a log scale.

No ethanol was detected at 0 h after feeding but its concentration increased to a maximum of 5.4 mM at 1 h. After this maximum ethanol concentration steadily decreased, falling below the detection limit by 4 h (Figure 2A). No lactate was detectable at 0 and 0.5 h, and a numerical maximum of 2.7 mM was observed at 1 h, after which concentrations decreased to below the detection limit by 4 h (Figure 2B). After feeding, total VFA concentration increased from 69 mM at 0 h to its numerical maximum of 123 mM after 3 h with the values at 2 and 2.5 h not significantly differing from the numerical maximum (Figure 2C). The molar proportion of acetate decreased from 68 to 62% over the first 1.5 h post feeding and then recovered toward the 0 h level after 3 h from feeding (Figure 2D). Propionate proportion showed the opposite pattern in time and significantly increased from 16% to its numerical maximum of 22% at 1.5 h, after which it declined to a proportion not significantly different from the 0 h level (Figure 2E). The proportion of butyrate showed a different pattern with a steady increase after feeding, from 11% at 0 h to a peak of 15% at 6 h after feeding (Figure 2F). Propionate proportion tended to be greater (0.61 ± 0.35%) and ethanol concentration tended to be lower (−1.3 ± 0.6 mM) for LSO compared to CON. No effects or tendencies for diet (*P* > 0.536) to affect the other dissolved metabolites assessed were observed and no time × diet interaction was observed for any of the dissolved metabolites.

**Figure 3:**
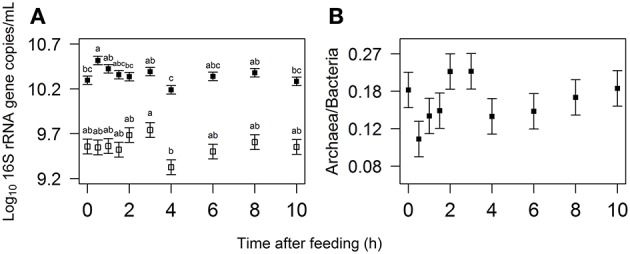
**(A)** Log_10_ transformed bacterial (closed squares) and archaeal (open symbols) 16S rRNA gene concentration and **(B)** back-transformed archaea to bacteria ratio in the rumen over the first 10 h after feeding with a *y*-axis that is plotted on a log scale. Values represent least square mean (LSM) ± standard error, with different letters indicating significant differences in time (*P* < 0.05).

In response to feeding the bacterial concentration (log_10_ 16S rRNA gene copies per mL rumen fluid) increased significantly from 0 to 0.5 h, and at 4 h was significantly lower than at 0.5, 1, and 3 h (Figure 3A). Linseed oil supplementation increased the bacterial concentration (10.4 vs. 10.3 log_10_ 16S rRNA gene copies/mL; *P* < 0.001); no time × diet interaction was observed (*P* = 0.899). The archaeal concentration (log_10_ 16S rRNA gene copies/mL) tended to be affected by time after feeding (*P* = 0.077), with the 3 and 4 h observations significantly different from each other (*P* = 0.014). No diet effect (*P* = 0.385) and time × diet interaction (*P* = 0.941) on the archaeal concentration were observed. The ratio of archaea to bacteria varied from 0.11 to 0.22 (Figure 3B) and tended to be affected by time from feeding (*P* = 0.089), with no significant differences between time points (*P* ≥ 0.138). Neither a diet effect (*P* = 0.611) nor a time × diet interaction was observed (*P* = 0.934) for the archaea to bacteria ratio.

### Hydrogen and methane emission

Average emission rates of H_2_ during daytime, which was from morning feeding at 6 a.m. to afternoon feeding at 4 p.m., were 33.0 and 34.3 mmol/h for CON and LSO fed cows, respectively (Table S3). Average emission rates of H_2_ overnight, which was from afternoon feeding at 4 p.m. to morning feeding at 6 a.m., were 28.3 and 28.1 mmol/h for CON and LSO diets, respectively. Average daytime CH_4_ emission rates were 1.12 and 1.07 mol/h and average overnight CH_4_ emission rates 1.05 and 1.02 mol/h for CON and LSO diets, respectively.

**Figure 4:**
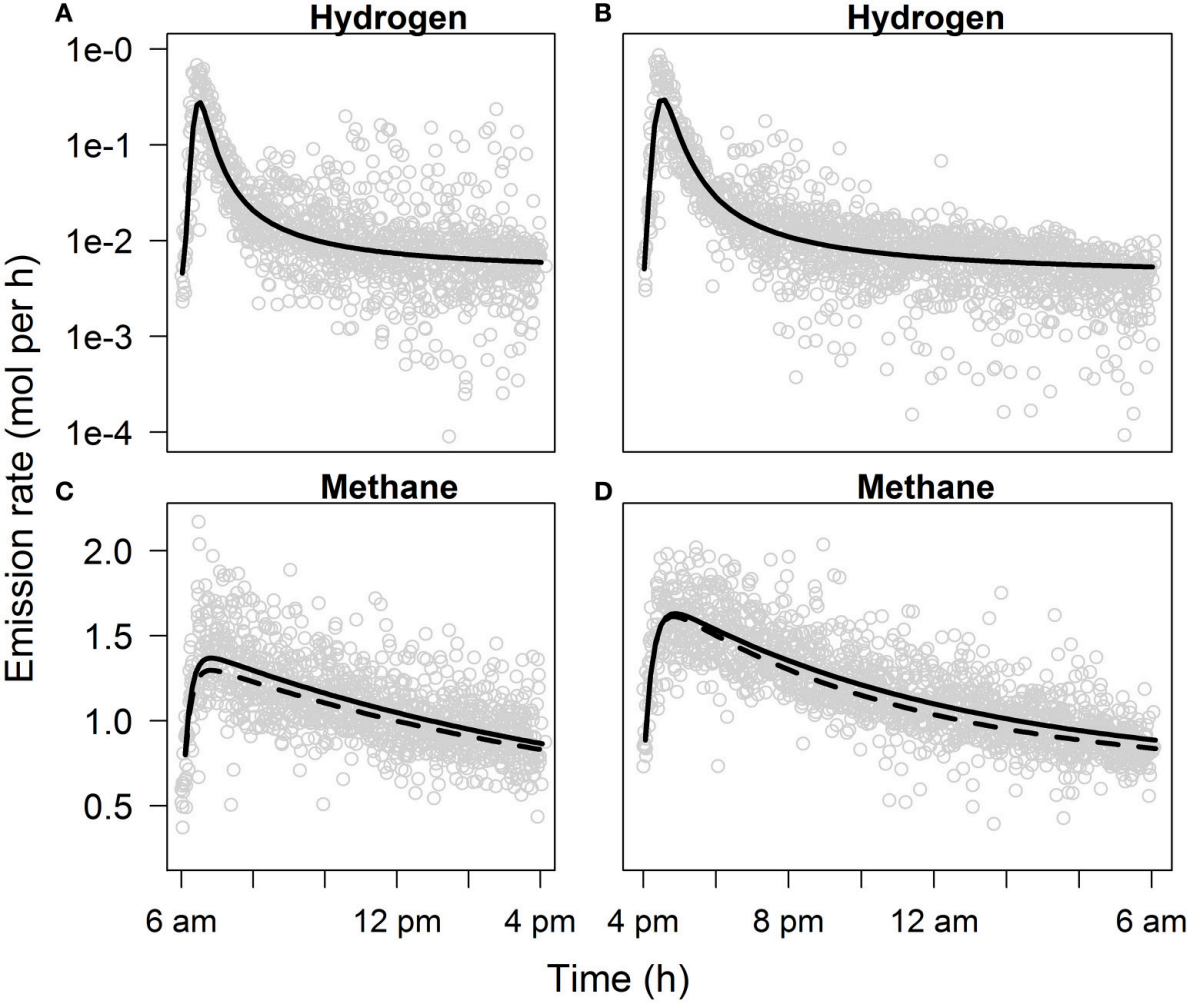
**Gas emission rates as a function of time from feeding**. The graph shows **(A)** daytime (from morning feeding at 6 a.m. to afternoon feeding at 4 p.m.) and **(B)** overnight (from afternoon feeding at 4 p.m. to morning feeding at 6 a.m.) back-transformed H_2_ emission rate plotted on a log scale predicted with a hyperbolic model, and **(C)** daytime and **(D)** overnight CH_4_ emission rate predicted with a double exponential model. H_2_ emission rate was not affected by dietary treatment, CH_4_ emission rate was affected by dietary treatment (solid line for control diet, dashed line for linseed oil diet). See Table S4 for model parameters.

The hyperbolic model fitted best to the log_10_ transformed H_2_ emission rate on AIC (Table S4). The double exponential model appeared to be insufficiently capable of fitting the sharp peak in H_2_ emission rate (result not shown). As substantial scattering of measurement points appeared after peak emission, modeling the H_2_ emission rate of the daytime and overnight periods with residual variance functions improved the model fit. The selected hyperbolic model showed an increase in H_2_ emission rate from ~5 to 200 mmol/h in 0.5 h after feeding and then decreased to basal level (Figures 4A,B). No diet effect was observed on any of the parameters of the best-fit models for daytime and overnight H_2_ emission.

Based on AIC, daytime and overnight emission rate of CH_4_ were best described by the double exponential model. The CH_4_ emission rate increased by about a factor two from ~0.7 to 1.5 mol/h in 0.8 h after feeding (Figures 4C,D). On AIC, best-fit models for CH_4_ emission resulted in parameters that were significantly affected by diet, indicating decreased CH_4_ emission from LSO fed cows. For daytime CH_4_ emission, β_2_ was affected by diet (difference CON − LSO, 0.08 ± 0.04; Table S4), whereas for overnight CH_4_ emission rate, β_4_ was affected by diet (difference CON − LSO, 0.02 ± 0.01).

### Microbial composition

Bacteria (80.8 ± 7.8% of the 16S rRNA sequences) were represented by 787 OTU whereas the archaea (18.6 ± 7.7% of the 16S rRNA sequences) were represented by 68 OTU. Of the 75 different genus-level phylogenetic groupings (72 for bacteria and 3 for archaea) that the 855 OTU could be summarized to, six dominant groupings represented a major proportion of the bacteria (71.1 ± 4.7% of the bacterial 16S rRNA sequences) and one grouping the archaea (93.5 ± 2.4% of the archaeal 16S rRNA sequences). These seven major genus-level phylogenetic groupings could be annotated to either the family (Succinivibrionaceae;genus-NotAnnotated [g-NA; similarly, fg-NA indicates family and genus could not be annotated, and ofg-NA indicates order, family and genus could not be annotated], Ruminococcaceae;g-NA and Christensenellaceae;g-NA) or genus level (*Ruminococcus, Butyrivibrio, Prevotella* and *Methanobrevibacter*). A summary of the relative abundances of the genus-level phylogenetic groupings is given with respect to both sampling time (Figure S2) and diet (Figure S3).

**Figure 5:**
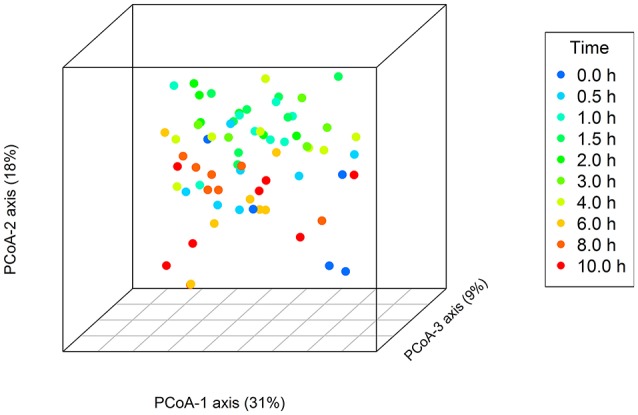
**Principal Coordinate Analysis of samples at the OTU level using weighted unifrac distances, with samples labeled by time point as indicated by the key**.

Principal Coordinate Analysis (PCoA) of the OTU-level data did not show any clear clustering of the samples with respect to either distinct time points or diet (Figure 5). The time points 0, 6, 8, and 10 h however were generally located to the bottom half of the PCoA-2 axis (18% of total variation), and the 1–4 h time points to the top. The 0.5 h time points were more centrally located along the PCoA-2 axis. No factors explaining variation could be identified for the separation of the samples on the PCoA-1 axis (31% of total variation). In line with the time point localization along the PCoA-2 axis, PERMANOVA indicated a difference in the microbial composition between the “low” (0, 6–10 h) and “high” (0.5–4 h) metabolite concentration categories (*P* < 0.001). Besides a few tendencies for significance, only the 1.0 and 1.5 h time points were significantly different from the 8 h time point (Table S5). PERMANOVA also indicated an effect of diet on microbial composition (*P* = 0.024), as well as a period × diet effect (Table S6). The period × diet as well as inherent cow variation in the rumen microbiota, may have limited the appearance of the diet effect in the PCoA plot.

**Figure 6:**
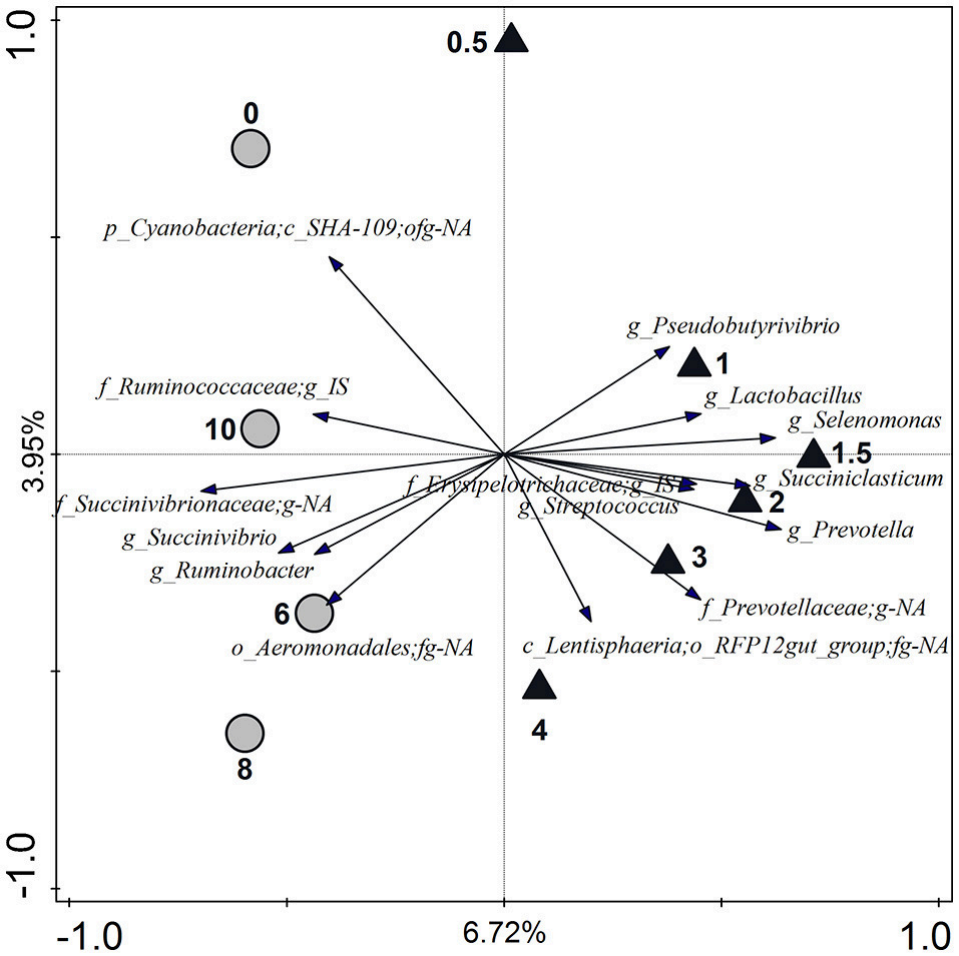
**Redundancy analysis triplot showing the relationship between the top 15 genus-level phylogenetic groupings of the OTUs explaining the variance with time**. The canonical axes are labeled with the percentage of the total variation accounted for with respect to the explanatory variable time. Time points (0–10 h) are indicated relative to the ruminal concentration of metabolites being either high (total VFA + lactate + ethanol > 90 mM or peaks in *P*_H_2__ and *P*_CO_2__, triangles) or low (all other concentrations, circles). Arrow length indicates the variance that can be explained by the parameter time, with the perpendicular distance of the time points to the arrow indicating the relative abundance of the genus-level phylogenetic grouping. Arrow labels indicate the taxonomic affiliation of genus-level phylogenetic groups, with the level [i.e., kingdom (k), phylum (p), class (c), order (o), family (f), or genus (g)] and taxon (as defined by the Silva 16S rRNA database) that the groups could be reliably assigned to. For example “g_*Prevotella*” represents an OTU reliably assigned to the *Prevotella* genus, whereas “p_Cyanobacteria;c_SHA-109;ofg-NA” was reliably assigned to the class SHA-109 but the order, family, and genus could not be annotated (NA). IS, Incertae Sedis.

**Figure 7:**
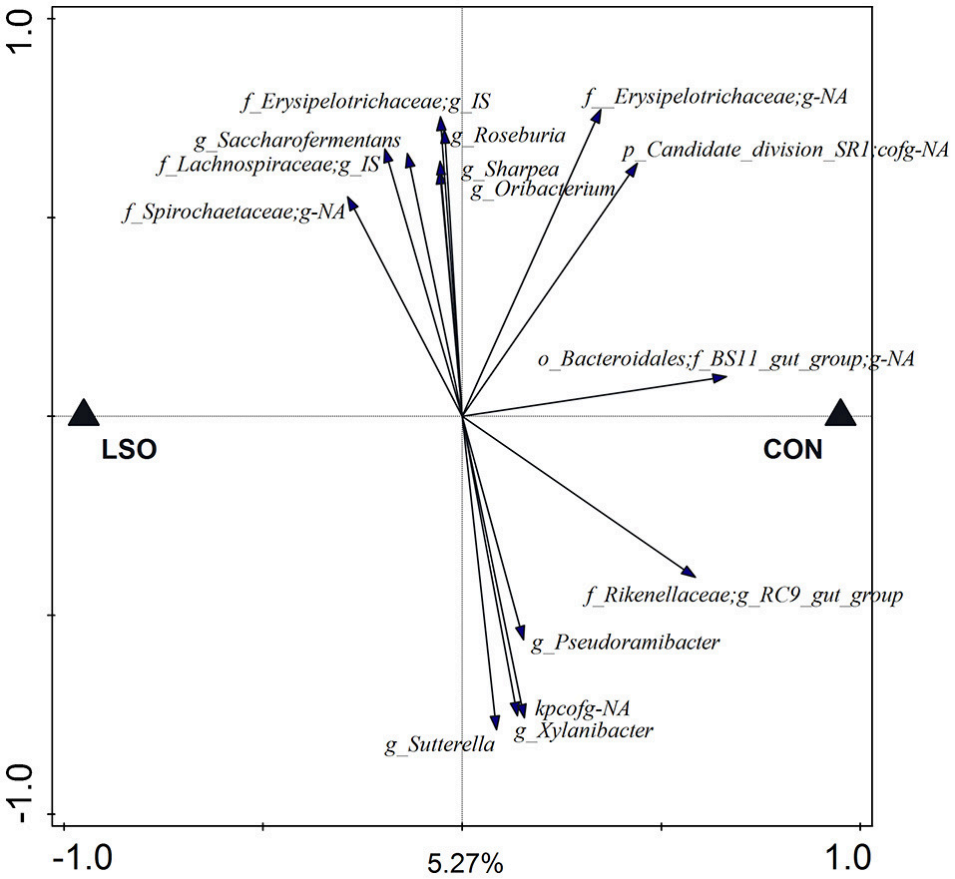
**Redundancy analysis triplot showing the relationship between the top 15 genus-level phylogenetic groupings of the OTU explaining the variance with diet [control (CON) or linseed oil (LSO)]**. The canonical axis is labeled with the percentage of the total variance accounted for by the explanatory variable diet. Arrow length indicates the variance that can be explained by diet; distance and labels are as previously described in Figure 6. IS, Incertae Sedis.

Time points separated along the first canonical axis of the RDA by the “low” and “high” time point categories (Figure 6). *Pseudobutyrivibrio, Lactobacillus, Selenomonas, Succiniclasticum, Streptococcus*, and *Prevotella* genera appeared to be associated with the “high” time point category, along with some genus-level phylogenetic groupings that could only be annotated to the family (Prevotellaceae and Erysipelotrichaceae Incertae Sedis) or order level (Lentisphaeria RFP12 gut group). Ruminococcaceae Incertae Sedis, *Succinivibrio*, and *Ruminobacter* genera appeared to be associated with the “low” time point category, along with some genus-level phylogenetic groupings that could only be annotated to the family (Succinivibrionaceae), order (Aeromonadales), or class level (Cyanobacteria SHA-109). Many of the genus-level phylogenetic groupings also differed further in terms of the time points where their relative abundance was highest (Figure 6). The nine genus-level phylogenetic groupings for the “high” time point category had high relative abundances at the following times after feeding: *Pseudobutyrivibrio* (0.5–1.5 h), *Lactobacillus* (1–2 h), *Selenomonas* (1.5 h), *Succiniclasticum* (1.5 h), Erysipelotrichaceae Incertae Sedis (1.5 h), *Streptococcus* (1.5 h), *Prevotella* (1.5 h), Prevotellaceae;g-NA (1.5–3 h), and Lentisphaeria RFP12 gut group (4 h). The six genus-level phylogenetic groupings for the “low” time point category had high relative abundances at the following times after feeding: Cyanobacteria SHA-109;ofg-NA (0–0.5 h), Aeromonadales;fg-NA (8 h), *Ruminobacter* (8 h), *Succinivibrio* (8 h), Succinivibrionaceae:g-NA (8–10 and 0 h), and Rumincoccaceae Incertae Sedis (10 and 0 h). Of the variation in the relative abundance of genus-level phylogenetic groupings that were best explained by diet, only two groupings appeared to have high relative abundance associated with one of the diets (Figure 7). The Bacteroidales BS11 gut group and the Rikenellaceae RC9 gut group had a positive association with CON, and were therefore negatively associated with the LSO.

## Discussion

To our knowledge, this is the first comprehensive study that has identified the diurnal profiles of gaseous and dissolved metabolites (including lactate and ethanol) and the microbiota composition in the rumen, along with associated respiration chamber measured H_2_ and CH_4_ emission rates. It is unique that these diurnal profiles were mapped with at least 10 time points during the first 10 h after feeding, and the obtained data illustrated the importance of frequent sampling during the first few hours after feeding. This insight is important when developing an integrated understanding of the dynamics of rumen microbial fermentation, and its implications for the production of H_2_ and CH_4_.

### Gaseous metabolites

The basal level of *P*_H_2__ observed in the present study is similar to the lower bound values of 0.1–0.6 μM (1 · 10^−4^–8 · 10^−4^ bar of *P*_H_2__ according to Henry's law) for the rumen of cattle and sheep as reported in the review of Janssen (2010). Hegarty and Gerdes (1999) suspected rumen *P*_H_2__ in cattle and sheep to be rarely higher than 1 · 10^−2^ bar, which applies to all our observations except for the one at 0.5 h after feeding. The maximum of *P*_H_2__, however, is still in line with Smolenski and Robinson (1988) who reported a H_2_ spike of 10–20 μM (1–3 · 10^−2^ bar of *P*_H_2__) in steers that lasted for 30 min after feeding. Moate et al. (1997) reported 0.66 and 0.76 bar of *P*_CO_2__ and 0.31 and 0.22 bar of *P*_CH_4__ in the rumen of dairy cows, before and after an hour of active grazing, respectively. These absolute values are higher than observed in the present study, but the increased *P*_CO_2__ and decreased *P*_CH_4__ in response to feed consumption is similar.

The increase in H_2_ emission rate, of which the magnitude reflects the increase observed in ruminal *P*_H_2__, is similar to the profile shown by Rooke et al. (2014) where a H_2_ emission peak from a forage-concentrate fed steer appeared shortly after feeding. The H_2_ yield in Rooke et al. (2014), however, appeared to be higher (0.11 mol/kg DM) than observed in the present study (0.04 mol/kg DM). Olijhoek et al. (2016) observed the lowest average dairy cow H_2_ emission of 6 mmol/h over the hour before feeding, and the highest average H_2_ emission of 134 mmol/h over the first hour after feeding for their control diet. Veneman et al. (2015) observed maximum H_2_ emission rate of about 125 mmol/h from dairy cows during the first hour after feeding for control and linseed diets. These hourly averages are generally in line with the results in this study (minimum 5 mmol/h; maximum 200 mmol/h; peak at 0.5 h). The fitted CH_4_ emission profiles in the present study are in line with Brask et al. (2015) who reported the highest average hourly emission from dairy cows in the second hour after feeding, whereas the highest average hourly emission was observed in the third hour after feeding by Olijhoek et al. (2016). Rooke et al. (2014) and Olijhoek et al. (2016) observed an increase in CH_4_ emission by a factor of two after feeding, which is similar to the increase observed in the present study. Given the CH_4_ emission rate and DMI, the CH_4_ yield in the present study is 22.6 g/kg of DM, which is comparable to the CH_4_ yields for dairy and beef cattle reported by Veneman et al. (2015) for control and linseed oil diets, van Gastelen et al. (2015) and the mixed diet of Rooke et al. (2014) ranging from 21.4 to 25.0 g/kg of DM.

The fitted emission rate of both gases showed a rapid increase after feeding, whereas residual variance of the H_2_ emission rate increased after the peak emission, in particular for the daytime period (Figure 4). Upward scattering may have been caused by delayed feed intake as not all cows ingested their feed within the same period of time (Figure S1), while downward scattering might have resulted from decreased activity of cows. Although the selected non-linear model visually appears to properly estimate the average emission rate in time, in future experiments on rumen fermentation dynamics it might be useful to give cows access to feed only during the first few hours after feed delivery. Data following a non-skewed distribution would be particularly helpful when making inference on effects such as diet.

The increase of ruminal *P*_H_2__ and *P*_CO_2__ at the expense of *P*_CH_4__, and the peak in H_2_ emission rate shortly after feeding can be explained by microbial fermentation of rapidly degradable feed components, yielding H_2_ and CO_2_. Archaea in turn use the H_2_ and CO_2_ released from fermentation to produce CH_4_, which is reflected in the peak in CH_4_ emission rate that follows the peak in H_2_ emission rate (at 0.8 and 0.5 h, respectively). Increased archaeal production of CH_4_ relative to microbial fermentation in response to feed intake may have caused the recovery of *P*_H_2__, *P*_CO_2__, and *P*_CH_4__ toward the basal level, as observed from 1 h after feeding. The coincidence of a sharp peak in H_2_ emission and a relatively weak increase in CH_4_ emission, followed by a steeper decline in H_2_ emission compared with CH_4_ emission after the peak emission, is in line with Olijhoek et al. (2016). These patterns suggest that for the observed range of *P*_H_2__, the archaeal enzymes available became saturated with H_2_ as a methanogenic substrate and operated at their maximum rate.

### Fermentation dynamics and microbiota

The sharp peak in H_2_ emission shortly after feeding is associated with the microbial degradation of rapidly fermentable feed contents such as sugars (e.g., Leedle et al., 1982) as many different rumen micro-organisms rapidly utilize these. Apart from the Cyanobacteria, no genus-level phylogenetic grouping of OTU had a high relative abundance at the 0.5 h time point. The lack of further specific association with the 0.5 h time point might indicate that almost all micro-organisms can swiftly use rapidly degradable soluble substrates, resulting in no single species being more abundant than the others at this time.

Several of the genus-level phylogenetic groups in the rumen were positively associated with the time points that were within 1–3 h after feeding: *Pseudobutyrivibrio, Lactobacillus, Selenomonas, Succiniclasticum*, Erysipelotrichaceae *Incertae Sedis, Streptococcus, Prevotella*, and Prevotellaceae;g-NA (Figure 6). The majority of these genera are known for their ability to promptly utilize non-structural carbohydrates. *Lactobacillus* was most abundant between 1 and 2 h after feeding, which coincided with the appearance of their major fermentation end product lactate.

Species of *Ruminobacter* and *Succinivibrio* are involved in starch degradation (Bryant and Small, 1956; Anderson, 1995), and their abundance between 6 and 10 h suggests that starch utilization is a key activity of the planktonic rumen bacteria at this stage (Figure 6). This may occur at this time due to release of (or increased access to) internal plant cell components as the structural carbohydrates are broken down by fibrolytic microbes. Bacteria degrading complex structural carbohydrates however were probably under-represented in our study as only rumen fluid was sampled.

The ecological role of the Aeromonadales;fg-NA and Ruminococcaceae *Incertae Sedis* genus-level phylogenetic groups, which were positively associated with the 4–10 h, and the 6–10 and 0–0.5 h time points, respectively, could not be identified due to the limited functional annotation of these groups. It is possible though that these phylogenetic groups are involved in cross-feeding of secondary metabolites released by the action of other microbes that colonize the feed particles. In line with this, Leedle et al. (1986) reported that the changes in time that occurred in the carbohydrate composition in the rumen of steers were not always consistent with the predicted scheme of fermentation of carbohydrates such as cellulose/hemicellulose, pectin, starch, and soluble sugars.

In line with an early increase in metabolic activity, reflected by the H_2_ production spike shortly after feeding, bacterial concentration increased to its highest value at 0.5 h (Figure 3). As bacterial proliferation may not occur rapidly enough to solely explain this increase, microbes might have migrated from the rumen particulate matter to rumen fluid in response to the freshly ingested feed, which temporarily increased the bacterial concentration in the fluid. The significant decline observed at 4 h is in line with the previous findings of Leedle et al. (1982), who also observed a minimum in direct bacterial counts at 4 h after feeding a 77% forage diet to steers. The bacterial decline from 3 to 4 h is likely to be associated with decreased metabolic activity, as evidenced by the significant decrease in total VFA concentration also from 3 to 4 h after feeding (Figure 2). Since the bacterial concentration did not consistently increase until 2 h after feeding (whereas the total VFA concentration did) other processes may have been counteracting an increase in the bacterial concentration in the rumen fluid, such as adherence of bacteria to feed particles.

Archaeal concentration followed a similar pattern to the bacterial concentration, except no increase was observed at 0.5 h after feeding. Rumen methanogenic archaea do not directly utilize feed but only fermentation products such as H_2_, which explains why the peak in emission of CH_4_ appeared after the peak in H_2_ emission (0.8 vs. 0.5 h). The less steep decline of CH_4_ emission after its peak compared to H_2_ emission suggests that the archaeal enzymes are saturated with H_2_ producing CH_4_ still close to their maximum rate. The increased bacterial concentration and unaffected archaeal concentration at 0.5 h after feeding resulted also in a numerically decreased archaea to bacteria ratio. Wallace et al. (2014) found that the archaea to bacteria ratio in the rumen of beef cattle may be an indicator of CH_4_ yield per amount of feed. In the present study, the dynamics of the archaea to bacteria ratio and *P*_CH_4__ were qualitatively similar, both having a minimum at 0.5 h after feeding (Figures 1, 3). This would imply that the archaea to bacteria ratio is associated with the amount of CH_4_ produced relative to the total active metabolism, or in other words resembling the CH_4_ yield per amount of feed degraded.

The *P*_H_2__ up to 2.2 · 10^−2^ bar may thermodynamically inhibit hydrogenase catalyzed NADH oxidation in rumen bacteria (e.g., Van Lingen et al., 2016). In this thermodynamic state, the metabolism oxidizes NADH back to NAD^+^ by generating more reduced fermentation products (e.g., Counotte and Prins, 1981; McSweeney et al., 1994; Fischbach and Sonnenburg, 2011). This explains why increased proportions of propionate at the expense of acetate were observed, and why lactate and ethanol appeared in response to feeding (Figure 2). These findings are therefore consistent with such a shift in metabolism being driven by a decreased NAD^+^ to NADH ratio.

Increased propionate and decreased acetate proportion in response to feeding is in line with several reports (e.g., Brask et al., 2015; Hatew et al., 2015) that observed the lowest acetate to propionate ratio at 2 h after feeding in dairy cattle. The peak in lactate concentration appeared to be lower and later in response to feeding than in Counotte and Prins (1981), who observed 16, 29, and 16 mM of lactate at 15, 30, and 60 min after feeding 6 kg of concentrates. The lower amount of rapidly degradable carbohydrates in the 70:30 roughage to concentrate ratio diets used in this study may explain this difference.

Ethanol concentration in the rumen has not been widely measured *in vivo*, but was found to accumulate in the rumen of cattle and sheep after overfeeding with readily fermentable carbohydrates (Allison et al., 1964). The highest ethanol concentration occurred 1 h after feeding and was associated with among others the genus *Pseudobutyrivibrio* (Figure 6), which has been reported to include a species capable of producing ethanol (Kopečný et al., 2003). The decrease in ethanol and lactate concentrations after 1 h, combined with the decrease of the propionate proportion in favor of acetate from 1.5 h, suggest that NADH oxidation was no longer strongly inhibited. This is also consistent with the observation that the *P*_H_2__ declined in combination with a decreased pH. Besides elevated concentrations of VFA, lactate will also contribute to a decrease in pH despite its relatively low concentration. This is because it is a stronger acid than acetate, propionate or butyrate. From 1 h after feeding, the pH in the rumen remained significantly decreased for several hours. The decrease in pH until 2–3 h after feeding followed the increase in total VFA concentration (Figures 1, 2). Decreased pH counteracts the inhibition of NADH oxidation caused by increased *P*_H_2__ (Van Lingen et al., 2016), which alleviates the inhibition of NADH oxidation from 1 h after feeding.

The DMI in the present study was close to the feed intake of an average cow in the Netherlands (Bannink et al., 2011), despite the fact that the cows were in late lactation. In general, we believe that the DMI, rather than the lactation stage of cows, affects rumen fermentation diurnal profiles. For example, Sutton et al. (1988) evaluated three levels of intake at three ratios of hay to concentrate and at three stages of lactation (weeks 1–8, 9–16, and 17–36). This study reports that VFA molar proportions were not affected by stage of lactation, whereas a larger feed intake level did increase molar proportion of propionic acid, and decreased that of acetic acid. Early lactation cows in general have a higher DMI than late lactation cows. A higher DMI increases the amount of substrate available for fermentation and may result in more extreme *P*_H_2__ peaks and a stronger inhibition on NADH oxidation, and in turn more lactate and ethanol production and a lower acetate to propionate ratio. Hence, intake level explains more variation in rumen fermentation processes than stage of lactation. In view of an average feed intake level for a dairy cow achieved in our study, we consider the results to be indicative for the rumen of an average dairy cow.

### Effects of linseed oil supplementation

The lack of effect of LSO on emission of H_2_ is in line with Veneman et al. (2015), who also did not observe a difference in H_2_ emitted from control and linseed treated dairy cows with 2.2 and 6.2% crude fat, respectively. Similarly, Troy et al. (2015) did not observe an effect on H_2_ emitted from beef cattle when feeding a control and rapeseed cake treated diet with 2.7 and 5.4% crude fat, respectively. In the present study, linseed oil decreased CH_4_ emission rate and tended to decrease ruminal *P*_CH_4__. This is consistent with the study of Martin et al. (2016) where decreased CH_4_ emission was observed in response to increasing linseed supply with hay and corn silage based diets fed to dairy cattle. In contrast Veneman et al. (2015) did not observe a significant effect of linseed treatment on emission of CH_4_ in the two experiments they performed. In a meta-analysis, Patra (2013) indicated CH_4_ emission to be affected by the amount of C18:3, a major component in linseed oil, but demonstrated that the CH_4_-suppressing effect might be more marked with high concentrations of non-fiber carbohydrates in diets. However, Livingstone et al. (2015) did not find a decrease in CH_4_ emission upon linseed supplementation regardless of the ratio of grass silage to corn silage in the diet of dairy cattle. Various non-fiber carbohydrates may yield different VFA proportions, which makes it difficult to explain the CH_4_-suppressing effect of C18:3 in detail. Biohydrogenation of unsaturated fatty acids also serves as a H_2_ sink, but only has a minor contribution to the decrease in CH_4_ production (Czerkawski, 1986). Furthermore, decreased CH_4_ production may have resulted from decreased H_2_ production, because oils are not fermented and their degradation does not yield H_2_.

A tendency for an increased molar proportion of propionate for the LSO diets, is in line with the study of Li et al. (2015) who observed a decreased acetate to propionate ratio 3 and 6 h after feeding in steers fed linseed compared with control diets. Moreover, Martin et al. (2016) reported an increased propionate proportion with increased linseed supply in dairy cattle. Changes in the proportion of VFA may therefore be a component of the C18:3 mode of action, as propionate proportion tended to be increased in the present study.

The archaea concentration was unaffected by diet but bacterial concentration increased. The biological significance of this increase is not clear however as no corresponding increase of total VFA occurred in LSO fed animals. Other studies, however, differ in their reports of the effect of linseed oil on microbial concentrations. Veneman et al. (2015) reported that the bacterial concentration associated with the solid phase tended to be decreased upon linseed oil supplementation, whereas the bacteria concentration in the fluid was not significantly affected. Yang et al. (2009) found that increased proteolytic bacteria at the expense of cellulolytic bacteria occurred rather than increased total viable bacteria concentration upon linseed oil supplementation in dairy cattle. Differences observed in results from the present study may be related to the ruminal site of sampling as the rumen is not a completely homogeneous environment. The cranial ventral sac, which was the sampling site in this study, is known to have a higher total VFA concentration, lower pH, and differ in its microbiota and activity compared to the central rumen sacs (Martin et al., 1999; Wang et al., 2016a). Another possible explanation is that the increased concentration of planktonic bacteria with linseed oil was caused by a decreased number of bacteria able to colonize the feed particles (Duval et al., 2004). This would also partly explain the lack of a concurrent increase in total VFA concentration. Sampling of the rumen solid contents should be considered to investigate a possible reduction in fiber colonization. In the present study, however, this was not possible as the required opening of the fistula would have abolished the possibility to determine detailed headspace gas profiles after feeding.

The linseed oil supplementation appeared to have a limited impact on the rumen microbiota composition and the metabolite concentrations in our study, consistent with other studies (Li et al., 2015; Veneman et al., 2015). In the present study, diet also did not induce changes in archaeal species diversity. For both the metabolite concentrations and the microbiota composition, time from feeding clearly explained more variation than diet. Differences in the degradation rates of various types of carbohydrates may explain the observed temporal variation in metabolite concentration (Leedle et al., 1982) and microbiota composition (e.g., Rooke et al., 2014; Li et al., 2015; Wang et al., 2016b). Therefore, an experimental approach with contrasts in carbohydrate degradation rate, rather than contrasts in the amount of dietary fat, might have revealed even more about the microbial metabolic dynamics in the rumen.

Despite the limited effect of the linseed oil on the rumen microbiota, the linseed oil still decreased ruminal CH_4_ emission. The variation in the rumen microbiota associated with the period × diet, might be due to the various grass silage batches used in this experiment. The crude fat and WSC fractions of the grass silage were 32 and 95 g/kg of DM in period 1 and 26 and 81 g/kg of DM in period 2, respectively, which might explain the period × diet effect. It may have also prevented diet specific effects in the rumen microbiota composition analysis from being detected. This is evidenced by the various genus-level phylogenetic groupings in the RDA triplot that had no substantial association with either of the experimental diets. The Bacteroidales BS11 gut group was most clearly negatively associated with the LSO diet. Despite the indication of its role in hemicellulosic sugar fermentation (Solden et al., 2017), no cultured representative is available for this taxon. It is, therefore, not clear by which mechanism the linseed oil supplementation would decrease their relative abundance. C18:3 may be toxic to this taxon, as has been previously reported for various other rumen bacteria (Maia et al., 2007). Despite a tendency for decreased CH_4_ emission in the present study, the 16S rRNA gene sequence based microbial community analysis did not indicate any correlation between abundance of methanogens and dietary treatment. This absence of correlations does, however, not necessarily imply that the activity of metabolic pathways of archaea were unaffected. Shi et al. (2014) studied transcription of the archaeal *mcrA* gene, encoding one of the key enzymes in methanogenesis, in the sheep rumen and identified a discrete set of rumen methanogens whose methanogenesis pathway transcription profiles correlated with CH_4_ emission.

### Summary of main findings

In conclusion, time after feeding appeared to explain more variation in diurnal pattern of rumen metabolite concentrations and microbial composition than the CON and LSO diets. The large variation observed in diurnal patterns of rumen metabolites, the substantial increase of *P*_H_2__ rapidly after feeding followed by the occurrence of shifts in fermentation toward ethanol, lactate, and propionate at the expense of acetate, supports the key role of the redox state of NAD in rumen fermentation. This highlights the importance of including diurnal dynamics in rumen fermentation studies to improve understanding of VFA and CH_4_ production. The findings of this study also give insight into the key control points of rumen microbial metabolism, providing future opportunities to develop novel sustainable approaches to reduce the ecological footprint of ruminant livestock production.

## Author contributions

HV, JV, SV, BV, CP, HS, and JD designed the experiment. HV, SV, BV performed the sampling. HV, JV, and JE performed the laboratory analyses. HV statistically analyzed the metabolite and qPCR profiles. JE and ES statistically analyzed the microbial community composition. AB, HS, CP, and JD supervised the work. HV and JE wrote the manuscript. All authors read and approved the final manuscript.

## Funding

The contribution of AB was financed from the project Low Emission Animal Feed financed by the Dutch Ministry of Economic Affairs (the Hague, the Netherlands), and by the Product Board Animal and the Dutch Dairy Board (Zoetermeer, the Netherlands).

### Conflict of interest statement

The authors declare that the research was conducted in the absence of any commercial or financial relationships that could be construed as a potential conflict of interest.
