# Supplementary material for: Diurnal Dynamics of Gaseous and Dissolved Metabolites and Microbiota Composition in the Bovine Rumen
Source: Front Microbiol. 2017 Mar 17;8:425. doi: 10.3389/fmicb.2017.00425 (PMC5355475; doi:10.3389/fmicb.2017.00425)
Supplement: Supplementary file 1 [file DataSheet1.docx]

Supplementary Material

Diurnal dynamics of gaseous and dissolved metabolites and microbiota composition in the bovine rumen

Henk J. van Lingen^1,2,*^, Joan E. Edwards^1,3^, Jueeli D. Vaidya^1,3^, Sanne van Gastelen^1,2^, Edoardo Saccenti^4^, Bartholomeus van den Bogert^1,3,#^, André Bannink^5^, Hauke Smidt^3^, Caroline M. Plugge^3^, Jan Dijkstra^2^

^1^Top Institute Food and Nutrition, Wageningen, Netherlands

^2^Animal Nutrition Group, Wageningen University & Research, Wageningen, Netherlands

^3^Laboratory of Microbiology, Wageningen University & Research, Wageningen, Netherlands

^4^Systems and Synthetic Biology, Wageningen University & Research, Wageningen, Netherlands

^5^Animal Nutrition, Wageningen Livestock Research, Wageningen, Netherlands

#) Present address: BaseClear, Leiden, Netherlands.

*** Correspondence:** Henk van Lingen: [henk.vanlingen@wur.nl](mailto:henk.vanlingen@wur.nl)

# Supplementary Figures and Tables

## Supplementary Tables

**Table S1.** Ingredient composition (g/kg DM) of experimental concentrates without linseed oil (CON) and with linseed oil (LSO)

| Item | CON | LSO |
| --- | --- | --- |
| Soybean meal | 400 | 369 |
| Soybean meal, formaldehyde treated | 200 | 184 |
| Rapeseed meal | 100 | 92 |
| Rapeseed meal, formaldehyde treated | 100 | 92 |
| Sugar beet pulp | 119 | 109 |
| Sugarcane molasses | 40 | 37 |
| CaCO_3_ | 15 | 15 |
| NaCl | 8 | 8 |
| NaHCO_3_ | 2 | 2 |
| Trace mineral and vitamin mix^a^ | 8 | 8 |
| MgO | 7 | 7 |
| Cr_2_O_3_ | 2 | 2 |
| Linseed oil^b^ | 0 | 76 |

^a^ Research Diet Services, Wijk bij Duurstede, Netherlands. ^b^ Linagro, Lichtervelde, Belgium.

**Table S2.** Repeated-measures ANOVA *P*-values of time, diet, time × diet and period fixed effects and diet least square differences (LSD) for control diet minus linseed oil diet for partial pressure of gases in the headspace, pH, dissolved metabolite concentrations and 16S rRNA gene based microbial concentrations in rumen fluid.

| Metabolite | time | diet | time × diet | period | LSD ± SE^a^ |
| --- | --- | --- | --- | --- | --- |
| $P_{H_{2}}$ (bar) | <0.001 | 0.285 | 0.209 | 0.566 | 4.6∙10^-2^ ± 4.2∙10^-2^ |
| $P_{\mathrm{CO}_{2}}$ (bar) | <0.001 | 0.932 | 0.433 | 0.791 | 1.2∙10^-3^ ± 1.4∙10^-2^ |
| $P_{\mathrm{CH}_{4}}$ (bar) | <0.001 | 0.067 | 0.567 | 0.005 | 1.3∙10^-2^ ± 6.8∙10^-3^ |
| pH | <0.001 | 0.538 | 0.902 | <0.001 | 0.06 ± 0.09 |
| Total VFA (mM) | <0.001 | 0.536 | 0.811 | 0.126 | −2.6 ± 4.1 |
| Acetate (% of VFA) | <0.001 | 0.604 | 0.808 | 0.076 | 0.56 ± 1.00 |
| Propionate (% of VFA) | <0.001 | 0.057 | 0.783 | 0.038 | −0.61 ± 0.34 |
| Butyrate (% of VFA) | <0.001 | 0.970 | 0.536 | 0.255 | 0.02 ± 0.55 |
| Lactate (mM) | 0.089 | 0.804 | 0.732 | 0.771 | 0.1 ± 0.2 |
| Ethanol (mM)^b^ | <0.001 | 0.065 | 0.184 | 0.832 | 1.3 ± 0.6 |
| Bacteria (16S rRNA gene copies/mL) | <0.001 | <0.001 | 0.899 | 0.564 | −0.09 ± 0.02 |
| Archaea (16S rRNA gene copies/mL) | 0.077 | 0.385 | 0.941 | 0.165 | −0.06 ± 0.06 |
| Archaea:Bacteria | 0.089 | 0.611 | 0.934 | 0.272 | 0.03 ± 0.06 |

^a^ log_10_-transformed values for $P_{H_{2}}$, lactate concentration and bacterial and archaeal concentrations are shown.

^b^ results for best model, without random effect of cow, are shown; when data were fitted to the model that included both random effect of cow and a spatial correlation structure (Eq. 1), a second-best fit was obtained where the repeated measures covariance matrix converged to zero, *P*-values were <0.001 (time), 0.003 (diet) , 0.313 (time × diet) and 0.205 (period), respectively.

**Table S3.** Descriptive statistics of daytime (D; from morning feeding at 6 am to afternoon feeding at 4 pm) and overnight (N; from afternoon feeding at 4 pm to morning feeding at 6 am) average dry matter intake during chamber period (DMI; kg/portion) and hydrogen and methane emission rates (mmol/h and mol/h) for control (CON) and linseed (LSO) fed cows.

|  | Time | mean | SD | min | max |
| --- | --- | --- | --- | --- | --- |
| DMI - CON | D | 8.9 | 0.2 | 8.7 | 9.3 |
| DMI - LSO | D | 9.0 | 0.4 | 8.0 | 9.3 |
| DMI - CON | N | 9.1 | 0.2 | 8.7 | 9.3 |
| DMI - LSO | N | 9.1 | 0.2 | 8.7 | 9.3 |
| H_2_ – CON | D | 33.0 | 76.4 | 1.53∙10^-1^ | 624 |
| H_2_ – LSO | D | 34.3 | 76.9 | 9.02∙10^-2^ | 680 |
| H_2_ – CON | N | 28.3 | 80.7 | 9.32∙10^-2^ | 746 |
| H_2_ – LSO | N | 28.1 | 75.8 | 1.53∙10^-1^ | 864 |
| CH_4_ – CON | D | 1.12 | 0.27 | 0.44 | 2.17 |
| CH_4_ – LSO | D | 1.07 | 0.24 | 0.37 | 1.89 |
| CH_4_ – CON | N | 1.05 | 0.30 | 0.38 | 1.93 |
| CH_4_ - LSO | N | 1.02 | 0.30 | 0.21 | 1.85 |

**Table S4.** Parameter estimates (± SE), peak emission time ($t_{\mathrm{peak}}$ in h) and fit statistic of selected double-exponential (DE) and hyperbolic (HB) models for daytime (D; from morning feeding at 6 am to afternoon feeding at 4 pm) and overnight (N; from afternoon feeding at 4 pm to morning feeding at 6 am) log_10_-transformed hydrogen and methane emission rates (mol/h). $\beta_{1}$is the asymptote, $\beta_{2}$ is a dimensionless linear multiplier, $\beta_{3}$ and $\beta_{4}$ determine the increase and decline of gas emission after feeding, respectively; if applicable, $\beta_{n}=\delta_{n1}x_{n1}+\delta_{n2}x_{n2}$, with $\left[ \begin{matrix} x_{n1} \\ x_{n2} \end{matrix} \right]=\left[ \begin{matrix} 1 \\ 0 \end{matrix} \right]$ if diet is control and $\left[ \begin{matrix} x_{n1} \\ x_{n2} \end{matrix} \right]=\left[ \begin{matrix} 0 \\ 1 \end{matrix} \right]$ if diet is linseed, $\delta_{n1}-\delta_{n2}$ is the least square difference of the control and linseed diet effects parameters associated with $\beta_{n}$.

| Model | time | $\beta_{1}$ | $\beta_{2}$ | $\beta_{3}$ | $\beta_{4}$ | AIC | $t_{\mathrm{peak}}$ |
| --- | --- | --- | --- | --- | --- | --- | --- |
| Hydrogen | | | | | |  |  |
| DE^a^ | D | -2.13±0.09 | 2.94±0.29  2.46±0.22 | -3.98±0.45 | -0.87±0.10 | 907 | 0.49 |
| DE | N | -2.31±0.07 | 2.22±0.08 | -5.74±0.34 | -0.47±0.07 | 203 | 0.48 |
| HB | D | -2.36±0.06 | 18.80±3.10 | 13.70±2.57 | 1.82±0.14 | 789 | 0.49 |
| HB | N | -2.40±0.06 | 9.19±0.69 | 5.14±0.47 | 1.20±0.08 | 52 | 0.52 |
| Methane | | | | | |  |  |
| DE^b^ | D | 0.38±0.05 | 1.08±0.08  1.00±0.08 | −5.15±0.55 | −0.08±0.01 | −772 | 0.82 |
| DE^c^ | N | 0.52±0.04 | 1.13±0.04 | −4.18±0.57 | −0.12±0.01  −0.14±0.01 | −1173 | 0.87  0.84 |
| HB | D | 0.65±0.06 | 0.98±0.15 | 0.42±0.04 | 0.55±0.06 | −698 | 1.19 |
| HB | N | 0.25±0.04 | 1.39±0.03 | 0.19±0.02 | 0.25±0.03 | −1159 | 1.25 |

^a^ $\delta_{21}-\delta_{22}$ = 0.48±0.20, *P*-value = 0.015; ^b^ $\delta_{21}-\delta_{22}$ = 0.08±0.04, *P*-value = 0.036; ^c^ $\delta_{41}-\delta_{42}$ = 0.02±0.01, *P*-value = 0.015.

**Table S5.** Bonferroni corrected *P*-values from PERMANOVA pairwise comparisons applied on the weighted unifrac distance matrix to evaluate the effect of time on rumen microbiota composition. *P*-values < 0.05 (bold and italics) are considered as significant, and *P*-values < 0.10 (underlined and italics) indicate a tendency.

|  | 0 h | 0.5 h | 1 h | 1.5 h | 2 h | 3 h | 4 h | 6 h | 8 h |
| --- | --- | --- | --- | --- | --- | --- | --- | --- | --- |
| 0.5 h | 1 |  |  |  |  |  |  |  |  |
| 1 h | *0.081* | 1 |  |  |  |  |  |  |  |
| 1.5 h | *0.081* | 0.207 | 1 |  |  |  |  |  |  |
| 2 h | 0.540 | 1 | 1 | 1 |  |  |  |  |  |
| 3 h | 1 | 1 | 1 | 1 | 1 |  |  |  |  |
| 4 h | 0.297 | 1 | 1 | 1 | 1 | 1 |  |  |  |
| 6 h | 1 | 1 | 1 | 0.351 | 1 | 1 | 1 |  |  |
| 8 h | 0.162 | *0.072* | ***0.009*** | ***0.018*** | 0.198 | *0.090* | 0.684 | 1 |  |
| 10 h | 1 | 1 | 0.468 | 0.117 | 1 | 1 | 1 | 1 | 1 |

**Table S6.** Bonferroni corrected *P*-values from PERMANOVA pairwise comparisons applied on the weighted unifrac distance matrix to evaluate the effect of period × diet on rumen microbiota composition. *P*-values < 0.05 (bold and italics) are considered as significant, and *P*-values < 0.10 (underlined and italics) indicate a tendency.

|  | CON_P1 | CON_P2 | LSO_P1 |
| --- | --- | --- | --- |
| CON_P2 | *0.097* |  |  |
| LSO_P1 | ***0.020*** | 0.170 |  |
| LSO_P2 | 0.196 | 0.232 | 0.124 |

## Supplementary Figures

**Figure S1.** Feed intake pattern per cow, diet and/or period on experimental day 11 after morning feeding.

**Figure S2.** Taxonomic summary of the samples by time with the major phylogenetic groupings (annotated to the closest possible taxonomic level (family or genus)), indicated as follows: (a) Succinivibrionaceae, (b) Ruminococcaceae, (c) *Ruminococcus*, (d) *Butyrivibrio*, (e) Christensenellaceae, (f) *Prevotella* and (g) *Methanobrevibacter*.

**Figure S3.** Taxonomic summary of the samples by diet with the major phylogenetic groupings (annotated to the closest possible taxonomic level (family or genus)), indicated as follows: (a) Succinivibrionaceae, (b) Ruminococcaceae, (c) *Ruminococcus*, (d) *Butyrivibrio*, (e) Christensenellaceae, (f) *Prevotella* and (g) *Methanobrevibacter*.
