# Supplementary figures and images for: Diurnal Dynamics of Gaseous and Dissolved Metabolites and Microbiota Composition in the Bovine Rumen
Source: Front Microbiol. 2017 Mar 17;8:425. doi: 10.3389/fmicb.2017.00425 (PMC5355475; doi:10.3389/fmicb.2017.00425)

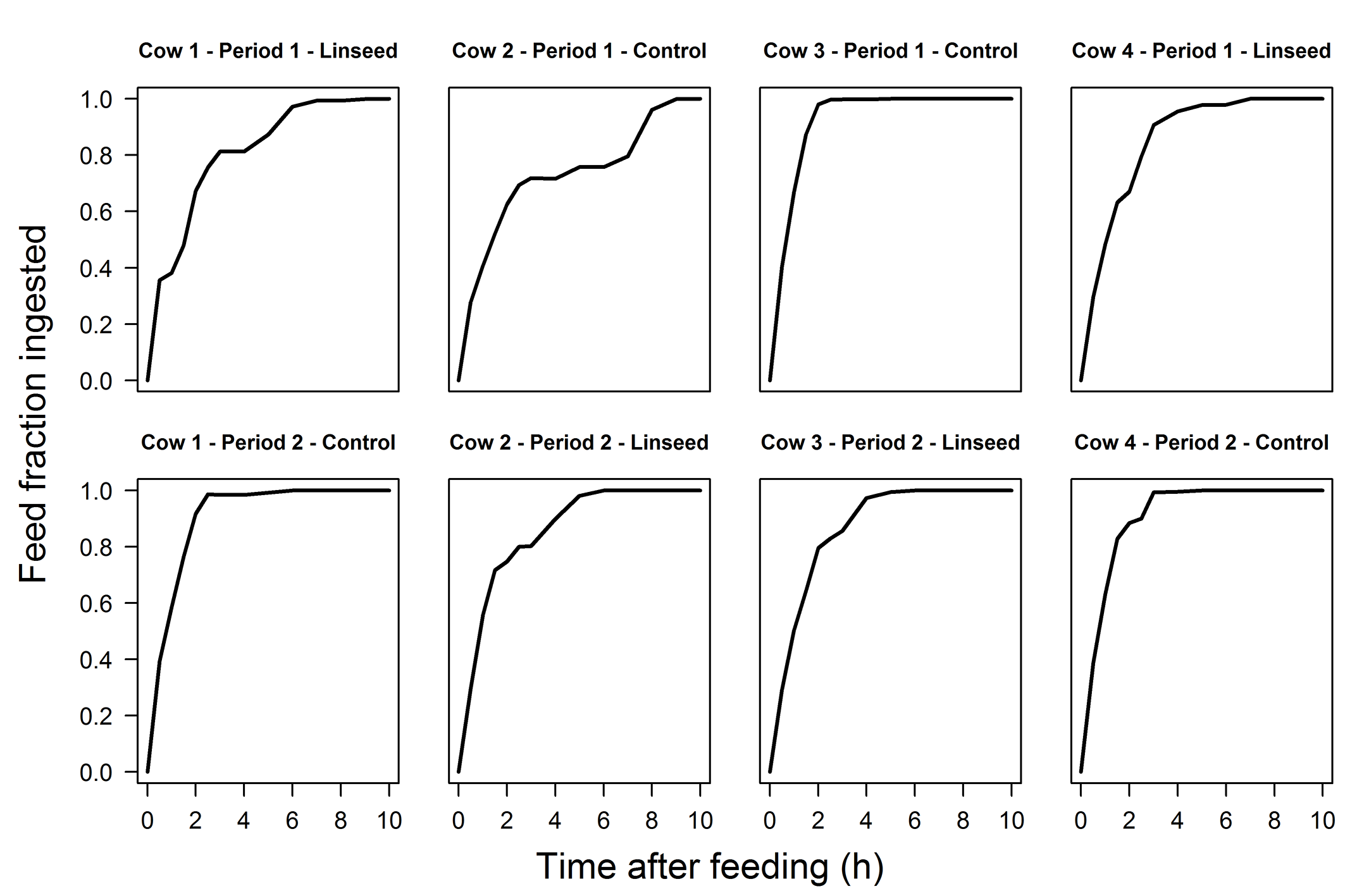

Supplement: Supplementary file 2 [file Image1.TIF]

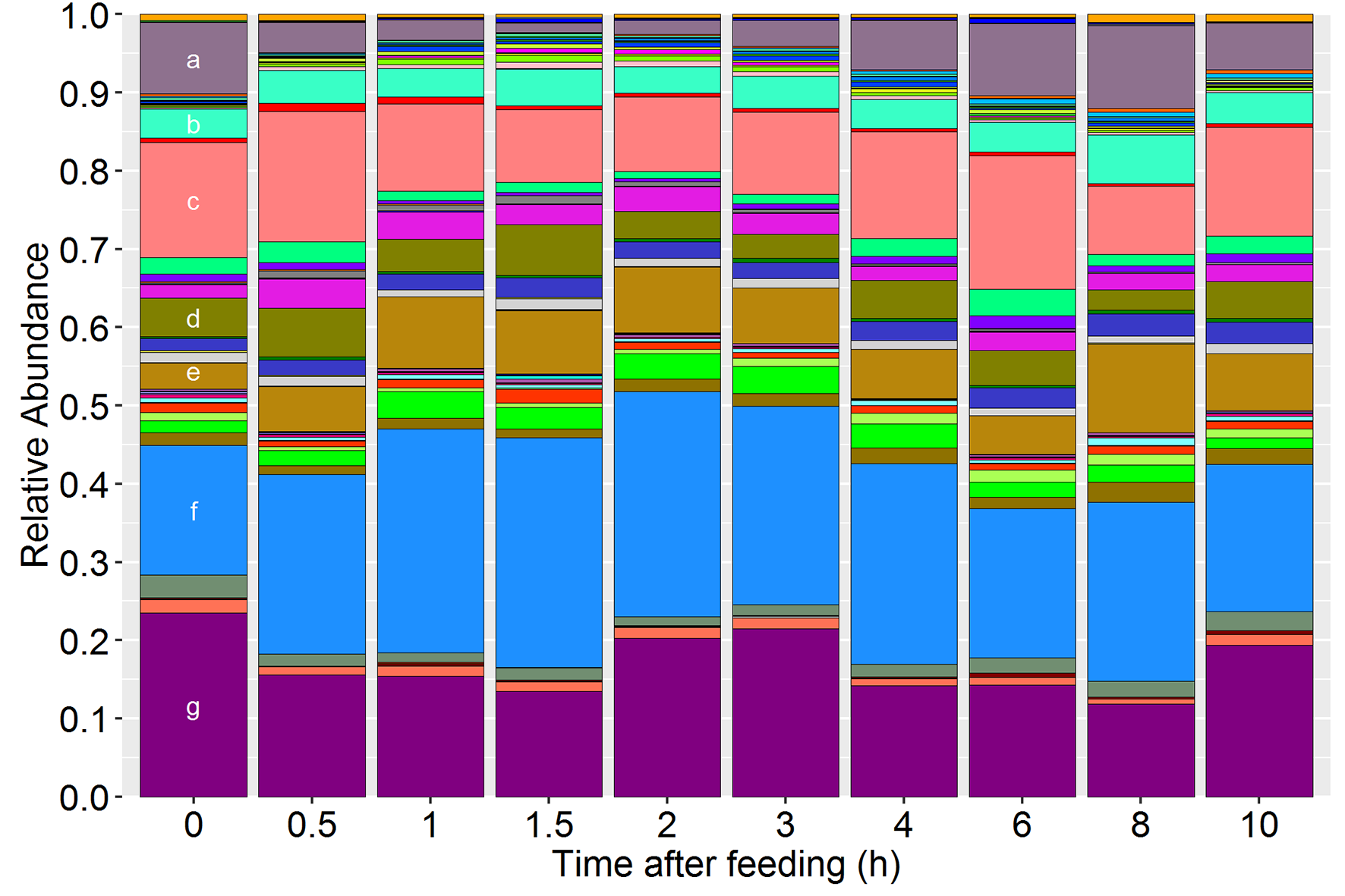

Supplement: Supplementary file 3 [file Image2.TIF]

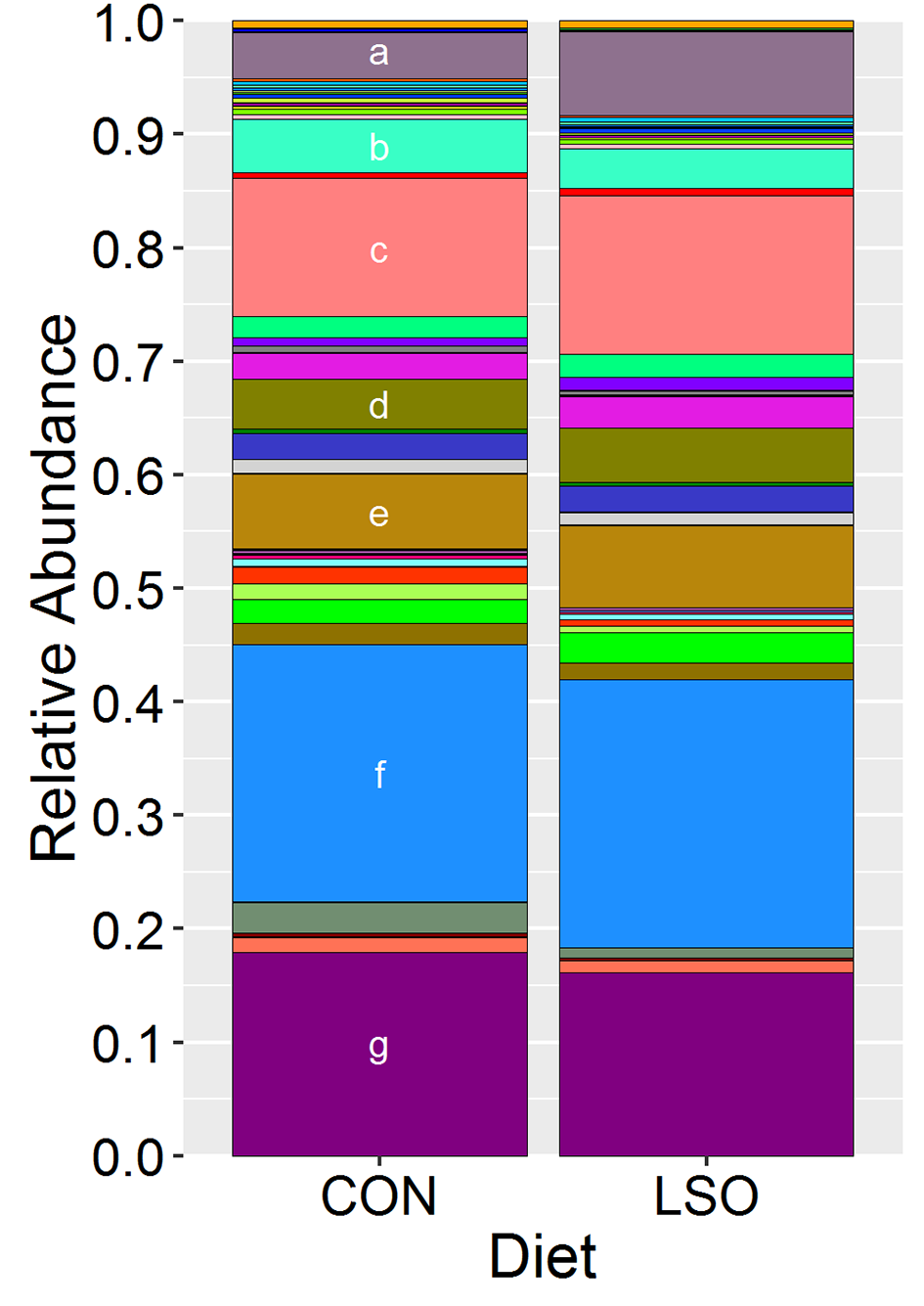

Supplement: Supplementary file 4 [file Image3.TIF]
